# *HLA* Diversity in the 1000 Genomes Dataset

**DOI:** 10.1371/journal.pone.0097282

**Published:** 2014-07-02

**Authors:** Pierre-Antoine Gourraud, Pouya Khankhanian, Nezih Cereb, Soo Young Yang, Michael Feolo, Martin Maiers, John D. Rioux, Stephen Hauser, Jorge Oksenberg

**Affiliations:** 1 Department of Neurology, University of California San Francisco, San Francisco, California, United States of America; 2 Histogenetics Inc., Ossining, New York, United States of America; 3 National Center for Biotechnology Information, National Library of Medicine, National Institutes of Health, Bethesda, Maryland, United States of America; 4 National Marrow Donor Program, Minneapolis, Minnesota, United States of America; 5 Université de Montréal Institut de Cardiologie de Montréal, Montréal, Quebec, Canada; Centro Cardiologico Monzino IRCCS, Italy

## Abstract

The 1000 Genomes Project aims to provide a deep characterization of human genome sequence variation by sequencing at a level that should allow the genome-wide detection of most variants with frequencies as low as 1%. However, in the major histocompatibility complex (MHC), only the top 10 most frequent haplotypes are in the 1% frequency range whereas thousands of haplotypes are present at lower frequencies. Given the limitation of both the coverage and the read length of the sequences generated by the 1000 Genomes Project, the highly variable positions that define *HLA* alleles may be difficult to identify. We used classical Sanger sequencing techniques to type the *HLA-A, HLA-B, HLA-C, HLA-DRB1* and *HLA-DQB1* genes in the available 1000 Genomes samples and combined the results with the 103,310 variants in the MHC region genotyped by the 1000 Genomes Project. Using pairwise identity-by-descent distances between individuals and principal component analysis, we established the relationship between ancestry and genetic diversity in the MHC region. As expected, both the MHC variants and the *HLA* phenotype can identify the major ancestry lineage, informed mainly by the most frequent *HLA* haplotypes. To some extent, regions of the genome with similar genetic or similar recombination rate have similar properties. An MHC-centric analysis underlines departures between the ancestral background of the MHC and the genome-wide picture. Our analysis of linkage disequilibrium (LD) decay in these samples suggests that overestimation of pairwise LD occurs due to a limited sampling of the MHC diversity. This collection of *HLA*-specific MHC variants, available on the *db*MHC portal, is a valuable resource for future analyses of the role of MHC in population and disease studies.

## Introduction

### 30 years of MHC genetics

The human major histocompatibility complex (MHC) is located in the short arm of chromosome 6p21. While the region contains only a small fraction of all human genes [1], it has been extensively studied due to its pivotal role in the immune response and the need for matching the human leukocyte antigen genes (*HLA*) between donor and recipient in allogeneic tissue and cell transplantation [2], [3]. For example, in addition to *HLA* typing performed for solid organ transplantation, *HLA* polymorphisms have been determined in more than 23 million unrelated donors worldwide in order to match patients in need of hematopoietic stem cell transplantation, [4]. Beyond transplantation, polymorphisms in the MHC region have been used as molecular markers for population genetics and studies of diseases and traits. In the past 30 years, no other region in the genome has provided more association signals with multifactorial traits, including autoimmune diseases [5]–[8], inflammatory and infectious diseases [9], cancer [10], adverse drug effects [11], [12], and behavioral traits such as mating [13], [14]. To assess *HLA* allelic diversity, these studies employed a broad range of methodologies from serology, restriction fragment length polymorphism, and microsatellites up to the latest generation of single nucleotide polymorphism (SNP) genotyping methods. In the most recent genome-wide association studies (GWASs), the high number of MHC-region SNPs included in the arrays and the great complexity of resulting association signals encouraged efforts to impute classical *HLA* alleles based on SNP profiles [15]. However, the extremely large number of known *HLA* alleles (unique gene sequences), currently over 8,000 for *HLA* class I genes and over 2,400 for *HLA* class II genes [16], [17], creates a formidable challenge when attempting to capture *HLA* alleles using genotypes derived from common SNPs, such as those typically included on GWAS arrays.

### Determining HLA polymorphisms in genomic reference samples

Building on the increasing feasibility of new generation sequencing methods, the 1000 Genomes Project provides a deep characterization of human genome sequence variation as a foundation for investigating the relationship between genotype and phenotype [18]. A goal of this project is to characterize over 95% of variants present (in genomic regions accessible to current high-throughput sequencing technologies) in 14 representative human populations from Europe, East Asia, South Asia, West Africa and the Americas. Whole genome sequencing is performed at low coverage, but at a level that should allow the genome-wide detection of most variants with frequencies as low as 1%, the classical threshold for definition of polymorphisms [18]. However, hundreds of well characterized *HLA* variants have frequency lower than 1%, and thousands of *HLA* haplotypes are present at even lower frequencies [19]. Because of the complexity of the exonic polymorphisms, several statistical methods are needed when calling *HLA* alleles from the sequence data [20], [21]. Higher coverage and longer read length that what the 1000 Genomes Project currently achieve, is required to positively identify all HLA alleles at all loci with an accuracy that compares to classical HLA typing experiments. The 1000 Genomes Project is nevertheless a primary reference dataset for modern genetic studies, including the SNP-based imputation of *HLA* alleles for disparate population and disease studies. In this report, we used sequence-based techniques to type alleles of the *HLA-A*, *HLA-B*, *HLA-C*, *HLA-DRB1* and *HLA-DQB1* genes in the available 1000 Genomes samples. This effort allowed the combined analysis of the 103,310 MHC SNPs made publicly available by the 1000 Genomes Project and the *HLA* alleles of these samples. While making these dataset available, we show that *HLA* alleles and MHC SNPs are extremely diverse in this dataset and highly specific to ancestral backgrounds. We also demonstrate that gathering *HLA* and SNP data on large numbers of samples worldwide increases the accuracy of HLA-SNP linkage disequilibrium (LD) estimations, revealing the *HLA* haplotype specificity of SNP variation. The availability of these *HLA* genotypes will promote analysis of the genomic architecture and immunobiology of this important super-locus at greater resolution than has heretofore been possible.

## Materials and Methods

### HLA typing by reference methods

The HLA typing assay was designed to capture the amino acid sequence of the Antigen Recognition Site (ARS) [22]. DNA samples were purchased from the Coriell Institute for Medical Research (Camden, NJ). The HLA typing data of 1,267 individuals related to the 1000 Genomes Project (Table 1) covers 14 populations encompassing 4 major ancestral groups. After specific PCR amplification, exons were sequenced by Sanger technique. The sequences were compared to available sequence information in the HLA allele database on exons 2 and 3 for class I and on exon 2 class II genes, therefore any polymorphism occurring in exon 4 of class I allele or exon 3 of class II gene was not investigated. Typing ambiguities between alleles were allowed since *HLA-A*, *HLA-B*, *HLA-C* gene products have identical sequences in exon 2 and exon 3 antigen recognition sites. Similarly, for class II genes, typing ambiguities occur if *HLA-DRB1*, *HLA-DQB1* gene products have identical sequences in exon 2 antigen recognition sites. (Appendix S1). The Allele Database version used in the report is IMGT 2.26.0 (Jul 2009), effective Feb 2010. Several Hapmap and CEPH samples were previously HLA typed [23], [24]. Confirmatory typing was performed when the typing of the five HLA loci were missing or ambiguous (12 samples). No discrepancies were found. The previously obtained HLA types were otherwise included. The public genotype calls for the 1000 Genomes sequence analysis were downloaded from 1000 Genomes servers (phase 1) for all available samples (See on-line resources [25]–[27]).

**Table 1:** Overview of the 1000 Genomes project samples typed for HLA genes.

*Code*	*Ancestry*	*Description of 1000 Genomes projects project Samples*	*Size*	*HLA and genomic variants*	*variants*
LWK	African	Luhya from Webuye, Kenya	90	87	97
YRI	African	Yoruba from Ibadan, Nigeria	90	38	88
ASW	American	African Ancestry from Southwest, USA	90	53	61
CLM	American	Colombian from Medellin, Colombia	70	60	60
MXL	American	Mexican Ancestry from Los Angeles-California, USA	89	55	66
PUR	American	Puerto Rican, Puerto Rico	70	55	55
CHB	East Asian	Han Chinese from Beijing, China	90	85	97
CHD	East Asian	Chinese from Denver-Colorado, USA	90	0	NA
CHS	East Asian	Han from south, China	100	100	100
JPT	East Asian	Japanese from Tokyo, Japan	91	80	89
CEU	European	Northern and Western European from Utah, USA	111	47	87
FIN	European	Finnish, Finland	100	93	93
GBR	European	British from England and Scotland, UK	96	89	89
TSI	European	Italian from Tuscany, Italy	90	90	98
TOTAL	4	14	1267	932	1080

Ibericos from Spain (n = 14) were genotyped in the KGP but were not available for *HLA* typing at the time of the project. Chinese Han from Denver were typed for *HLA* they are currently publically available for sequencing data.

### SNP genotype data from the 1000 Genomes project

The 103,310 MHC SNPs in the 1000 Genomes were extracted from the MHC (chr6: 28,866,528–33,775,446 See Table S1) [25]–[27]. Similar number of variants was extracted at random throughout the genome [28]. Additional variants were extracted in regions of the genomes with similar density of variants and similar recombination rate to the characteristics of the MHC region. Among the MHC variants, 6,040 MHC SNP previously genotyped in 800 African American controls [29], were used to compute linkage disequilibrium decay with distance by resampling datasets of various sample sizes. All coordinates refer to genome build HG19/GRCh37.

### Data availability on-line

The *HLA* genotype data of the present study is available online: The full specification of *HLA* alleles in the specified release of the *HLA* nomenclature [17] is provided on the *db*MHC portal (See on-line resources) at NCBI [30]. In addition, allele frequencies can be viewed online using tools developed in the anthropology and cell line components of the Histocompatibility Workshops [31](Figure S2, screen capture of the display [32]). Allele frequency tables are extremely sparse, reflecting the high diversity of *HLA* alleles for all loci and the limited sampling of the *HLA* alleles in 1000 Genome Project sample sets (Tables S2 and S3 for *HLA* allele naming convention used, also available online at dbMHC [32]).

## Results

### Ancestral diversity of the MHC in the 1000 Genomes samples

**Figure 1:**
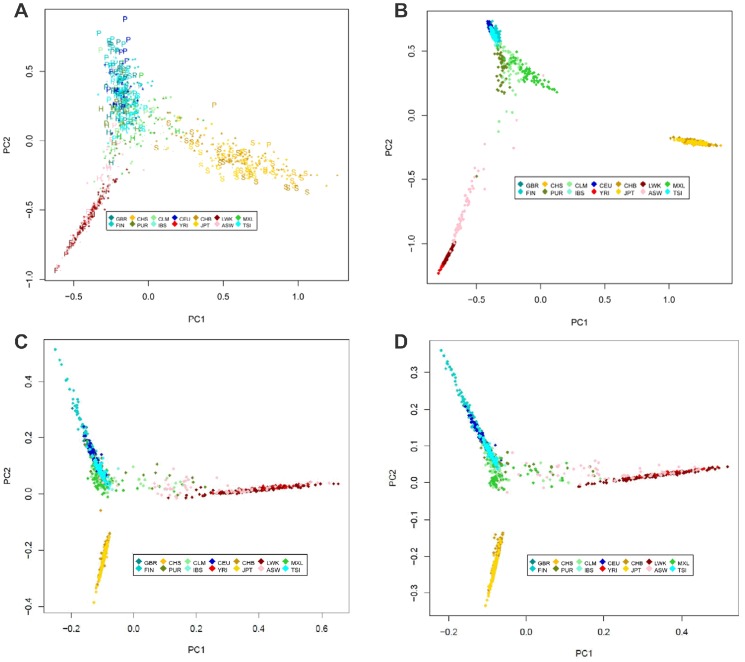
Principal Component analysis of the pairwise IBD distances between 1000 Genomes samples using MHC region marker (A), genome-wide markers (B), and using markers of regions with similar variants' density (C, chr9 : 116,750,000–121,650,000), with a recombination rate (D, chr9:800,000–5,700,000). (A) *The presence of the most frequent ancestry specific HLA haplotype in the samples of the 1000 Genomes project using MHC region markers*. Principal component analysis of the 103 K variants from the MHC region in the 1000 Genomes samples. PC1 captures 6.00% of total variance; PC2 captures 5.05%. The PCA analysis is based on publicly available SNPs. In order to integrate the SNP based information to the HLA allele information, individual spots are replaced by letters when a frequent *HLA* haplotype is predicted when the *HLA* typing is phased using *HLA* haplotype frequencies. The so called “frequent” haplotypes are defined in an ancestry specific manner: P for frequent *HLA* haplotypes in Europeans, S for frequent *HLA* haplotype in Asians, H for frequent *HLA* haplotype in Hispanics and F for frequent haplotype in Africans. The detailed list of the frequent haplotypes is presented in supplementary information. Frequent haplotypes and definition of overlap between ancestries were documented in a recent modeling effort for the development of haplobank. (B) *Principal Component analysis of the pairwise IBD distances between 1000 Genomes samples using genome-wide markers*. Principal component analysis of 100 K variants selected at random throughout of the genome in the 1000 Genomes samples. PC1 captures 55.16% of total variance PC2 captures 41.96%. The representation of distances computed from genome-wide SNPS clearly identifies samples of European, Asian and African ancestries. The results are consistent with self-declared ancestry and the admixed nature of several populations. There are however a few notable exceptions: NA20314 from south west African Americans (ASW) clusters with Mexicans (MXL), NA20291 from ASW clusters with LWK, and HG01108 from the Puerto Rican (PUR) who clusters with the majority of Africans Americans (ASW). In addition, four Columbians (CLM: HG01342, HG01390, HG01462, HG01551) and three African Americans (ASW: NA20278, NA20299, NA20414) cluster together away from their groups. These are also clustering far from their self-declared ancestry in the MHC centered analysis. This most likely reflects their genome-wide ancestry rather than a different ancestry of the MHC. (C) *Principal Component analysis of the pairwise IBD distances of 1000 Genomes samples using genome-wide markers of a region (chr9 : 116,750,000–121,650,000) with a variants' density that is similar to the* MHC *region*. Principal component analysis of 100 K variants selected at random throughout of the genome in the 1000 Genomes samples. PC1 captures 2.98% of total variance PC2 captures 1.56%. The representation of distances computed from genome-wide SNPS clearly identifies samples of European, Asian and African ancestries. PC1 and PC2 have been flipped to ease the comparison of the patterns in Figures 1A and 1B. (D) *Principal Component analysis of the pairwise IBD distances of 1000 Genomes samples using genome-wide markers of a region* (chr9:800,000–5,700,000) *with an avergage recombination rate that is similar to the* MHC *region*. Principal component analysis of 100 K variants selected at random throughout of the genome in the 1000 Genomes samples. PC1 captures 2.55% of total variance PC2 captures 1.57%. The representation of distances computed from genome-wide SNPS clearly identifies samples of European, Asian and African ancestries. PC1 and PC2 have been flipped to ease the comparison of the patterns in Figures 1A and 1B.

To focus on the ancestral information embedded in SNPs from the MHC, we compared the principal component analyses (PCA) of the Identity by Descent (IBD) distances between all individuals of the 1000 Genomes MHC dataset. IBD distances were computed using Beagle 2 [33] and averaged over ten runs. Both, the variants in the MHC region and an equal number of SNP variants randomly selected throughout the genome were used. The variants' density and recombination rate were computed from 1000 Genome data using Beagle (See web resources). We compared the IBD distances PCA analysis using the SNPs of the MHC region (Figure 1A), using the same number of SNPs randomly selected throughout the genome (Figure 1B). The MHC region has been also compare to other regions of the genomes with similar density of variant (Figure 1C) and similar recombination rate (figure 1D) (Additional examples and information in Figure S1 A–C)). As expected, the analysis shows that distances computed from genome-wide SNPs clearly identify samples of EuropeS1an, Asian and African ancestries as well as the admixed nature of several populations: ASW, PUR, CLM, and MXL (Figure 1B). Discordance between observed IBD ancestry and self-declared ancestry was seen for a handful of samples (Figure 1B legend).

When genetic similarity is computed using MHC SNPs only, the analysis clearly identifies the same three major ancestral lineages (Europeans, Asians, and Africans) (Figure 1A). Like regions with similar variants' density and recombination rate, MHC captures well the major ancestry backgrounds. However, more variability is observed within the MHC of the 3 major ancestries (Figure 1C and 1D), this is consistent with the selection of diversity driven by HLA molecules in a cumulative manner for class I and class II. Some individuals spread across the population hubs and display significant overlap (Figure 1A). This reflects the close relation between MHC polymorphisms and the migratory history of these populations [34], [35]. For example, in contrast to the genome-wide analysis, samples of African ancestry (YRI, LWK, and most of the ASW) overlap fully. African American samples (ASW) appears more split between European and African ancestries. It suggests that intra-group differences can rarely be differentiated from cross-ancestry sequence variation, at least for these populations. Thus, within the major ancestral backgrounds, SNP haplotypes can be shared between individuals of different populations. This observation further supports the empirical *HLA* compatibility of donor/recipient from distinct populations grounding international exchanges of allogeneic HSC donors. We conclude that the variability of the ancestral MHC signature may not be fully captured by the overall genome ancestral estimation potential, potentially weakening case control analyses due to stratification. For example, several African American samples whose genome-wide IBD distances indicate close relation to Africans cluster with the European groups in the MHC region-based analysis (NA19703, NA19707, NA19904, NA19921).

### Frequent HLA haplotypes in the 1000 Genomes samples

In order to integrate these results based on SNPs with the classical *HLA* typing in Figure 1A, we used *HLA* haplotype frequencies from the National Marrow Donor Program Registry to infer the phase of the most frequent *HLA* haplotypes represented in the dataset [19], [36]. *HLA* information was integrated to the PCA graphical display and *HLA* genotypes were phased using the haplotype frequencies [36]. Given the sample size, only frequent haplotypes were displayed (frequency >1%, as defined in “frequent” *HLA* haplotype modeling of haplobank [37]). When the statistical phasing of HLA alleles results in the presence of a frequent haplotype, letters were used as symbol at the PCA coordinate of the individual (“P” for European haplotypes, “H” for haplotypes frequent in Hispanics, “S” for haplotypes frequent in Asians and “F” for haplotypes frequent in Africans Listed in Table S4). Frequent haplotypes are found along the axis drawn by the PCA, which is consistent with frequent *HLA* haplotypes driving the IBD similarities within an ancestry. Interestingly, the analysis identified the presence of typical European *HLA* haplotypes in Asians: A*03:01∼B*35:01∼DRB1*01:01 (81% posterior phase probability NA185596 [38]) and A*01:01∼B*57:01∼DRB1*07:01 (88% posterior phase probability HG00708 [9]) Thus, a typical Asian SNP background associated with an *HLA* type is compatible with a mixed European Asian haploytpe, confirming the SNP background differences of conserved *HLA* haplotypes because. Therefore, even if only a few copies of the most frequent haplotypes are found in the 1000 Genome samples, and even if chromosomal phase is statistically estimated, it appears that this dataset will rapidly allow the in-depth analysis of haplotype specific variants of interest for both *HLA* allele imputation and *HLA* haplotype inference (Sup. Table S5, Imputation analysis limited to tag- SNP is suggestive of the existence of Haplotype specific SNPs).

### Linkage disequilibrium decay in the MHC

**Figure 2:**
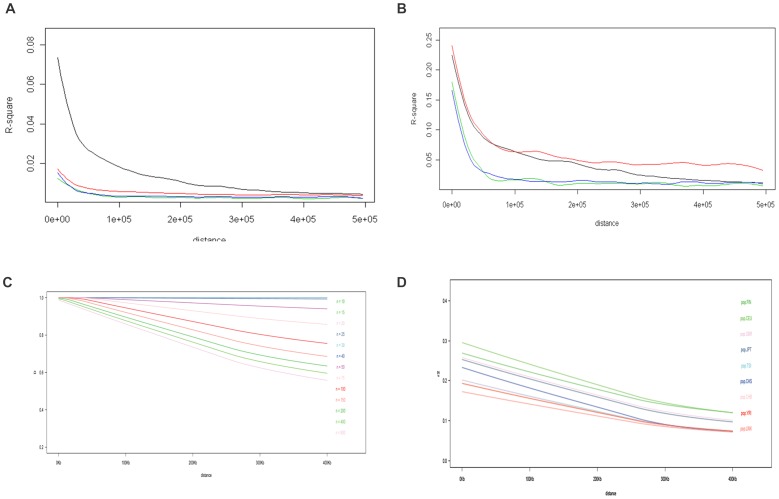
Across genomic region comparison of the Linkage Disequilibrium (LD) for variants with frequency lower than 5% (A), greater than 5% (B), and 90^th^ percentile of LD by D′ (C) and R^2^ (D) as a function of distance (kb) for various sample size as measure. (A) *Across genomic region comparison of the LD decay (R-Square) in the 1000 genome samples for variants whose frequency is lower than 5%*. (B) *Across genomic regions comparison of the LD decay (R-Square) in the 1000 genome samples for variants whose frequency is greater than 5%*. Chr6:28,850,000:33,750,000 (black) representing the MHC; Chr9:116,750,000:121,650,000 (green with similar variants' density as MHC used in Fig. 1C); chr9:800,000:5,700,000 (blue with similar recombination rate as MHC used in FIG. 1D), an additional control with similar variants' density chr8:9,400,000 = red (with similar variants' density as MHC), The plot is presented for 0–500 Kbp. In 2A, all markers are included in 2B only markers whose frequencies are greater than 5% are included, showing that the analysis is affected by low frequency variants which requires large sample size for accurate estimation. (C) *Average 90^th^ Percentile of pairwise linkage disequilibrium (D′) as a function of distance (kb) for various sample size*. (D) *Average 90^th^ Percentile of pairwise linkage disequilibrium (R2) as a function of distance (kb ranging from 0–400 Kb) for various sample sizes*. (C and D) The AAMS dataset consists of 405 African American controls and 594 African American individuals with multiple sclerosis (MS) typed at 6040 MHC SNPs using Infinium iSelect HD Custom Genotyping BeadChip (Illumina). After strict quality control for missingness <0.1% and minor allele frequency >5%, 3224 markers remained for analysis. A subset of n = 10 random control individuals was selected. Linkage disequilibrium (r2 and D′) was calculated between all pairs of SNPs (5,195,476 unique pairs) using Haploview software. All r2 and d′ estimates were sorted by distance between markers, and grouped into bins of 500 bases. The 90th percentile r2 and d′ were calculated within each bin. Locally weighted regression (Cleveland, W. S. (1981) LOWESS: A program for smoothing scatterplots by robust locally weighted regression (The American Statistician, 35, 54) was used to create a smooth regression line across the 90th percentile r2 and d′ measures. The line in the figure represents the median across 10 trials of re-sampling the n = 10 individuals. The same procedure was repeated for larger sample sizes (n = 15, 20, 25, 30, 40, 50, 75, 100, 150, and 200). For the largest sample sizes (n = 400 and n = 800), MS cases were included in the analysis. The Correlation between sample size and average LD measure at a distance of 400 kb is shown in Figure S3A and S3B in Supplementary material.

We followed on the analyses presented in Figure 1 in different regions of the genome comparing LD decay in segments with similar characteristics of the MHC (Figure 2A (rare variants) and 2B (variants >with frequent greater than 5%)). The results suggest that the MHC has a strong LD decay, but this decay depends also on the estimated frequencies of the variants affecting the comparison between regions (figure 2B). Then, in order to compare LD MHC configurations between populations we assessed the influence of the sample size on LD decay in the MHC region. By using the 90^th^ percentile of pairwise LD for a given distance between SNP variants, emphasis was placed on the strongest LD components, which are central to both genetic association studies and SNP-based imputation methods of *HLA* alleles. To evaluate samples sizes larger than those of the 1000 Genomes Project, high-density genotypes of the MHC in a large sample of African Americans from a previously published study were used [29]. Figures 2C and 2D show the 90^th^ percentile of D′ and r^2^ LD measures respectively for sample sizes ranging from N = 10 to N = 800 as a function of pairwise distance between MHC SNPs. As previously anticipated by Weiss and colleagues [39], sample size influences the estimation of LD: the smaller the sample size, the slower the LD decay with distance between markers. It demonstrates that for a sample size in the order of magnitude of those collected by the 1000 Genomes Project, LD in the MHC region is most likely overestimated. For low sample sizes, sampling fluctuations result in a drastic reduction of the haplotype diversity, which mimics a bottleneck effect. Such effect reduces the sample haplotype diversity compared to the source population haplotype diversity. It tends to inflate the estimation of the frequency of the sampled haplotype as compared to their real frequencies in the population and induces an overestimation of LD that diminishes in higher sample sizes. Such effect makes even more challenging the interpretation of genetic associations hitting the MHC because LD may extends further away from the primary signal than it appears from LD estimated with 1000 genome samples.

**Figure 3:**
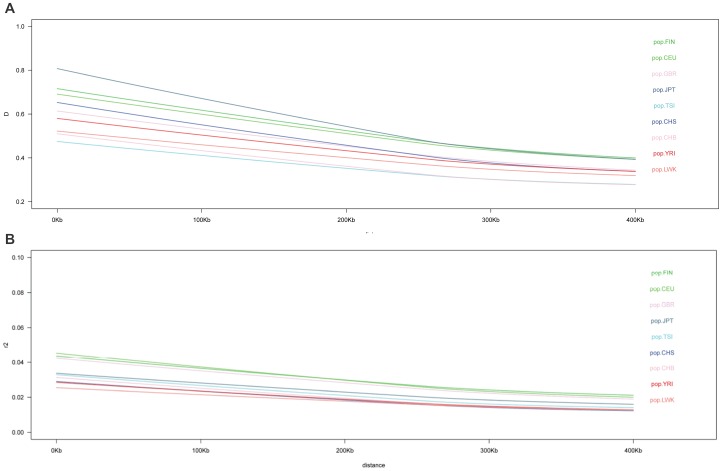
Across sample comparison linkage disequilibrium as a function of pairwise distance between SNPs for similar number of individual (n = 85) as measured by D′ (A) and R^2^ (B). (A) *Across sample comparison of Median of LD (D′) as a function of pairwise distance between SNPs for similar number of individual (n = 85)*. (B) *Across sample comparison of Median of LD (R2′) as a function of pairwise distance between SNPs for similar number of individual (n = 85)*. We resampled 85 unrelated individuals from the various populations of the 1000 Genomes in order to compare the LD decay pattern for a similar sample size. The figure shows the relation between the median percentile of pairwise LD measures according to the distance between the two markers between 0 and 400 Kb.

Next, we randomly sampled 85 unrelated individuals from nine of the1000 Genomes Project populations to directly compare the LD decay across the samples. Figure 3 displays the 90^th^ percentile of D′ (Figure 3A) and r^2^ (Figure 3B) LD measures (Y-axis) according to the distance between markers (X-axis) for nine populations of the 1000 Genomes project. The northern European populations (FIN and CEU) exhibit the highest LD along the MHC; British and Japanese samples have an intermediate LD. The Chinese (CHS and CHB), African (LWK and YRI), and surprisingly the Tuscani (TSI) samples have the lowest LD levels. These curves are influenced by both the genetic diversity of the most frequent haplotypes and the amount of recombination/drift occurring in the population history. In Tuscani and Chinese populations the most frequent *HLA* haplotypes are composed of frequent alleles. In Africans, *HLA* haplotypes are on average less frequent and more diverse. Given the density of genes in the MHC region and their functional relevance, long-range LD components can be involved in disease association signals; it also shows that using the analysis of samples of non-European ancestry can refine variants that may be causally involved.

## Discussion

We report the public availability of high resolution *HLA* typing in the samples of the 1000 Genomes Project and describe the ancestry specific content of *HLA* allele and SNP variant haplotypes of the MHC. The data complements the resource made available by the 1000 genomes project and other collaborative effort on those samples [23], [40]. The MHC region can be described as a “genome within the genome,” able to identify the ancestral history of the individual. However, the relative low sample size of the 1000 Genomes Project fails to properly reflect the full range of haplotype diversity and, consequently, the SNP-based analysis can overestimate the extent of LD patterns. Furthermore, the effect of sample size on LD depends on the baseline haplotype diversity and frequency distribution of the source population. While the difference observed between European and African populations is conservatively estimated, larger sample sizes would reduce this haplotype diversity truncation effect. The sample size effect is particularly strong on D′ measures of LD due to the apparently complete LD (D′ = 1 R^2^<1) that may generate sampling fluctuations that prevent interpretation of the D′ based LD decay comparison between populations.

### Large sample size are required to capture the haplotypic diversity of the MHC region

The availability of high resolution *HLA* typing information for the 1000 Genomes project dataset opens an array of possibilities for studying MHC polymorphisms and *HLA* alleles. It contributes to reducing the gap between large HLA registries, as illustrated by the recent publication of haplotype frequencies estimated from 2.9 million individuals [41], and the deep characterization of the human genome sequence diversity of the 1000 genomes project [18]. In addition to evolutionary studies of MHC haplotypes and HLA alleles, this HLA data will facilitate the training of the various SNP-based *HLA* imputation algorithms and the possibility to use the 1000 genome as reference samples for next-generation capture and sequencing of *HLA* genes. In order to illustrate the potential use of the public availability of the *HLA* gene typing with the 1000 Genomes sequencing data, we have explored the existence of SNP variants that can be used to indicate the presence of common *HLA* haplotypes (Sup. Table 2). Interestingly, many such variants seem to occur in the most common European *HLA* haplotype HLA-A*01:01∼HLA-B*08:01∼HLA-DRB1*03:01. The *HLA* haplotype HLA-A*3303∼HLA-B*4403∼HLA-DRB1*1302, which is common in Asians, also shows a high number of associated variants (r2>0.6, Sup. Table S4 and S5). Although *HLA* haplotype statistical phasing does not allow us to conclude that these are “tag-SNPs”, it adds further support to the examination of rare SNP variations embedded in long *HLA* haplotypes. Finally, we expect that the data will help to define the best reference and strategies for the use of SNPs to impute *HLA* alleles for population and disease studies.

### Supporting Information Legends

Supplemental data consists in: Figure S1 MHC region definition (Table S1), HLA Allele frequencies in the samples of the 1000 Genomes (Tables S2), HLA alleles grouped by similarities in the antigen recognition site (Table S3), Screen capture of the display of allelic frequencies in dbMHC for the 1000 genome populations (Figure S2), The most frequent ancestry specific HLA haplotypes (Tables S4), Please note that the V2 ‘old’ style HLA nomenclature and ARS “g” code were used in supplementary material, please refer to website for more up to date information and specification of HLA allele ambiguities strings. Variants associated with frequent haplotypes in Europeans (Tables S5), Correlation between sample size and r2 90th percentile in African American samples for marker of a pairwise distance of 1000 kb (Figure S3).

## Supporting Information

Figure S1 **MHC region definition.** A, Selection of the region by recombination rate et variants' density. B, chr16:86,750,000–91,650,000, a region on chromosome 16 with similar recombination rate as MHC shown in Figure 1D. **C**, chr16:74,200,000–79,100,000, a region on chromosome 16 with similar variants' density as MHC shown in Figure 1C.
(TIF)

Figure S2 **Screen capture of the display of allelic frequencies in dbMHC for the 1000 genome populations.** A, Homepage. B, Population selection. C, Data download.
(TIF)

Figure S3 **Correlation between sample size and the 90^th^ percentile of D′ (S3-A) and r2 (S3B) in African American samples for markers' pairs at a distance of 400 kb.**
(TIF)

Appendix S1 **Additional details for HLA typing protocol.**
(DOCX)

Table S1 **MHC region definitions.**
(DOCX)

Table S2 **HLA Allele frequencies in the samples of the 1000 Genomes.**
(DOCX)

Table S3 **HLA alleles grouped by similarities in the antigen recognition site.**
(DOCX)

Table S4 **The most frequent ancestry specific HLA haplotypes.**
(DOCX)

Table S5 **Variants associated with frequent haplotypes in Europeans.**
(DOCX)
