# Supplementary material for: HLA Diversity in the 1000 Genomes Dataset
Source: PLoS One. 2014 Jul 2;9(7):e97282. doi: 10.1371/journal.pone.0097282 (PMC4079705; doi:10.1371/journal.pone.0097282)
Supplement: Appendix S1 — Additional details for HLA typing protocol. (DOCX) [file pone.0097282.s004.docx]

**Appendix S1 Additional details for HLA typing**

Sequence-based typing (SBT) was used for typing HLA class I (HLA-A, -B, -C) and class II (DRB1 and DQB1) genes. Sequencing templates were produced by locus- or group-specific pairs of oligonucleotide primers from genomic DNA by Polymerase Chain Reaction (PCR). A total of 40 locus- and group- specific primers were used to amplify the target sequences.

Sanger cycle sequencing was carried out using BigDye V3.1 (Applied Biosystems) chemistry and ABI 3730xl capillary sequencer for base calling. The PCR product was treated with Exonuclease, and ethanol precipitation was used to clean-up post-sequencing reaction extension products. Class I sequencing primers were those locus-specific sequences for each locus in the intron/exon boundary regions to sequence the entire exons. All Class II PCR primers were tailed with M13 forward and T7 reverse sequences. Sequencing was performed on both strands.

When further resolution is required for the ambiguous combinations that were not distinguished by these groups, additional group-specific amplifications or group-specific sequencing primers were used.
