# Supplementary material for: HLA Diversity in the 1000 Genomes Dataset
Source: PLoS One. 2014 Jul 2;9(7):e97282. doi: 10.1371/journal.pone.0097282 (PMC4079705; doi:10.1371/journal.pone.0097282)
Supplement: Table S1 — MHC region definitions. (DOCX) [file pone.0097282.s005.docx]

**Table S1: MHC region definitions**

| MHC Sub-region | Number of variants | % of variants | SNPs | | Gene | | Position | |
| --- | --- | --- | --- | --- | --- | --- | --- | --- |
|  |  |  | From | to | From | to | From | To |
| Ext_CLASS_I | 11,180 | 11% | rs3130838 | rs1122947 | TRIM27 | MOG | 28,866,528 | 29,638,434 |
| CLASS_I | 42,718 | 42% | rs375984 | rs3131630 | ZFP57 | MICB | 29,644,502 | 31,485,354 |
| CLASS_III | 8,033 | 8% | rs4959079 | rs416352 | MCCD1 | NOTCH4 | 31,488,879 | 32,207,393 |
| CLASS_II | 29,906 | 29% | rs424232 | rs3129223 | AK123889 | HLA-DPB2 | 32,208,324 | 33,113,197 |
| Ext_CLASS_II | 9,403 | 10% | rs1003979 | rs1547668 | COL11A2 | MLN | 33,114,171 | 33,775,446 |
| Total | 103,310 | 100% | DB snp | | UCSC | | UCSC HG19/Build37 | |
