# Supplementary material for: HLA Diversity in the 1000 Genomes Dataset
Source: PLoS One. 2014 Jul 2;9(7):e97282. doi: 10.1371/journal.pone.0097282 (PMC4079705; doi:10.1371/journal.pone.0097282)
Supplement: Table S2 — HLA Allele frequencies in the samples of the 1000 Genomes. (DOCX) [file pone.0097282.s006.docx]

**Tables S2 HLA Allele frequencies in the samples of the 1000 Genomes**

| ***HLA Locus*** | ***Ancestry*** | ***African*** | | ***American*** | | | | ***East Asian*** | | | | ***European*** | | | |
| --- | --- | --- | --- | --- | --- | --- | --- | --- | --- | --- | --- | --- | --- | --- | --- |
|  | ***Code*** | ***LWK*** | ***YRI*** | ***ASW*** | ***CLM*** | ***MXL*** | ***PUR*** | ***CHB*** | ***CHD*** | ***CHS*** | ***JPT*** | ***CEU*** | ***FIN*** | ***GBR*** | ***TSI*** |
| ***HLA*A Alleles*** | A*0101g | 2 (1.11%) | 2 (1.67%) | 2 (1.89%) | 7 (5%) | 5 (4.24%) | 8 (5.71%) | 5 (2.78%) | 5 (2.78%) | 3 (1.5%) | 1 (0.55%) | 26 (17.33%) | 17 (8.5%) | 29 (15.1%) | 22 (12.22%) |
|  | A*0102 | 3 (1.67%) | NA | 1 (0.94%) | NA | NA | NA | NA | NA | NA | NA | NA | 1 (0.5%) | NA | NA |
|  | A*0201g | 30 (16.67%) | 5 (4.17%) | 15 (14.15%) | 26 (18.57%) | 19 (16.1%) | 23 (16.43%) | 30 (16.67%) | 15 (8.33%) | 20 (10%) | 19 (10.44%) | 55 (36.67%) | 68 (34%) | 59 (30.73%) | 41 (22.78%) |
|  | A*0202 | 7 (3.89%) | 3 (2.5%) | 9 (8.49%) | 3 (2.14%) | 1 (0.85%) | 3 (2.14%) | NA | NA | NA | NA | NA | NA | NA | NA |
|  | A*0203 | NA | NA | NA | NA | NA | NA | 5 (2.78%) | 10 (5.56%) | 12 (6%) | NA | NA | NA | NA | NA |
|  | A*0204 | NA | NA | NA | 2 (1.43%) | NA | 1 (0.71%) | NA | NA | NA | NA | NA | NA | NA | NA |
|  | A*0205g | 6 (3.33%) | 2 (1.67%) | NA | 3 (2.14%) | NA | 2 (1.43%) | 1 (0.56%) | 1 (0.56%) | NA | NA | 1 (0.67%) | NA | 2 (1.04%) | 1 (0.56%) |
|  | A*0206g | NA | NA | NA | NA | 8 (6.78%) | NA | 8 (4.44%) | 10 (5.56%) | 9 (4.5%) | 17 (9.34%) | 1 (0.67%) | NA | NA | NA |
|  | A*0207g | NA | NA | NA | NA | NA | NA | 4 (2.22%) | 17 (9.44%) | 30 (15%) | 2 (1.1%) | NA | NA | NA | NA |
|  | A*0210 | NA | NA | NA | NA | NA | NA | NA | NA | 1 (0.5%) | 1 (0.55%) | NA | NA | NA | NA |
|  | A*0211g | NA | 1 (0.83%) | NA | 3 (2.14%) | NA | 2 (1.43%) | NA | NA | NA | NA | NA | NA | NA | NA |
|  | A*0214 | 1 (0.56%) | NA | NA | NA | NA | NA | NA | NA | NA | NA | NA | NA | NA | NA |
|  | A*0217 | NA | NA | NA | NA | NA | NA | NA | NA | NA | NA | NA | NA | NA | 1 (0.56%) |
|  | A*0220 | NA | NA | NA | NA | NA | 2 (1.43%) | NA | NA | NA | NA | NA | NA | NA | NA |
|  | A*0222g | NA | NA | NA | 2 (1.43%) | NA | NA | NA | NA | NA | NA | NA | NA | NA | NA |
|  | A*0224 | NA | NA | NA | NA | NA | NA | NA | NA | NA | NA | NA | NA | 1 (0.52%) | NA |
|  | A*0301g | 7 (3.89%) | 15 (12.5%) | 6 (5.66%) | 19 (13.57%) | 11 (9.32%) | 16 (11.43%) | 7 (3.89%) | 1 (0.56%) | 3 (1.5%) | 1 (0.55%) | 15 (10%) | 57 (28.5%) | 25 (13.02%) | 24 (13.33%) |
|  | A*0302 | NA | NA | 1 (0.94%) | NA | NA | NA | 2 (1.11%) | NA | NA | NA | NA | NA | NA | 2 (1.11%) |
|  | A*0303N | NA | NA | NA | NA | NA | NA | NA | NA | NA | NA | 1 (0.67%) | NA | NA | NA |
|  | A*1101g | NA | NA | 1 (0.94%) | 2 (1.43%) | 8 (6.78%) | 8 (5.71%) | 45 (25%) | 52 (28.89%) | 52 (26%) | 13 (7.14%) | 9 (6%) | 9 (4.5%) | 17 (8.85%) | 9 (5%) |
|  | A*1102 | NA | NA | NA | NA | NA | NA | 2 (1.11%) | 8 (4.44%) | 8 (4%) | NA | NA | NA | NA | NA |
|  | A*1103 | NA | NA | NA | NA | NA | NA | NA | 1 (0.56%) | NA | NA | NA | NA | NA | NA |
|  | A*2301g | 10 (5.56%) | 13 (10.83%) | 6 (5.66%) | 5 (3.57%) | 1 (0.85%) | 6 (4.29%) | NA | NA | NA | NA | 4 (2.67%) | 1 (0.5%) | 2 (1.04%) | 6 (3.33%) |
|  | A*2402g | NA | NA | 4 (3.77%) | 22 (15.71%) | 18 (15.25%) | 17 (12.14%) | 32 (17.78%) | 31 (17.22%) | 38 (19%) | 68 (37.36%) | 7 (4.67%) | 11 (5.5%) | 14 (7.29%) | 22 (12.22%) |
|  | A*2403g | NA | NA | NA | 1 (0.71%) | 1 (0.85%) | 2 (1.43%) | NA | 1 (0.56%) | NA | NA | 1 (0.67%) | NA | NA | NA |
|  | A*2404 | NA | NA | NA | NA | NA | NA | NA | NA | 1 (0.5%) | 1 (0.55%) | NA | NA | NA | NA |
|  | A*2408 | NA | NA | NA | NA | NA | NA | NA | NA | 1 (0.5%) | NA | NA | NA | NA | NA |
|  | A*2420 | NA | NA | NA | NA | NA | NA | NA | NA | NA | 1 (0.55%) | NA | NA | NA | NA |
|  | A*2424 | NA | 1 (0.83%) | NA | NA | NA | NA | NA | NA | NA | NA | NA | NA | NA | NA |
|  | A*2501g | NA | NA | NA | 1 (0.71%) | 3 (2.54%) | NA | NA | NA | NA | NA | 4 (2.67%) | 1 (0.5%) | 6 (3.12%) | 1 (0.56%) |
|  | A*2601g | 1 (0.56%) | 3 (2.5%) | 1 (0.94%) | 3 (2.14%) | 4 (3.39%) | NA | 4 (2.22%) | 3 (1.67%) | 3 (1.5%) | 11 (6.04%) | 4 (2.67%) | 3 (1.5%) | 5 (2.6%) | 9 (5%) |
|  | A*2602 | NA | NA | NA | NA | NA | NA | NA | NA | NA | 2 (1.1%) | NA | NA | NA | NA |
|  | A*2603 | NA | NA | NA | NA | NA | NA | NA | NA | NA | 7 (3.85%) | NA | NA | NA | NA |
|  | A*2608 | NA | NA | 2 (1.89%) | NA | NA | NA | NA | NA | NA | NA | NA | NA | NA | 1 (0.56%) |
|  | A*2901g | 1 (0.56%) | NA | NA | 1 (0.71%) | NA | 2 (1.43%) | NA | 2 (1.11%) | NA | NA | NA | NA | NA | 2 (1.11%) |
|  | A*2902 | 13 (7.22%) | 2 (1.67%) | 5 (4.72%) | 7 (5%) | 4 (3.39%) | 10 (7.14%) | NA | NA | NA | NA | 4 (2.67%) | 2 (1%) | 11 (5.73%) | 2 (1.11%) |
|  | A*3001g | 16 (8.89%) | 11 (9.17%) | 9 (8.49%) | 3 (2.14%) | 5 (4.24%) | 1 (0.71%) | 8 (4.44%) | 6 (3.33%) | 3 (1.5%) | 1 (0.55%) | 1 (0.67%) | 2 (1%) | NA | 8 (4.44%) |
|  | A*3002 | 14 (7.78%) | 6 (5%) | 10 (9.43%) | 6 (4.29%) | 4 (3.39%) | 6 (4.29%) | NA | NA | NA | NA | 1 (0.67%) | NA | 2 (1.04%) | 3 (1.67%) |
|  | A*3004 | NA | NA | NA | NA | NA | 1 (0.71%) | NA | NA | NA | NA | NA | NA | 1 (0.52%) | 1 (0.56%) |
|  | A*3010 | NA | NA | NA | NA | NA | 1 (0.71%) | NA | NA | NA | NA | NA | NA | NA | NA |
|  | A*3018 | NA | NA | NA | NA | NA | NA | NA | 1 (0.56%) | NA | NA | NA | NA | NA | NA |
|  | A*3101g | NA | NA | 4 (3.77%) | 4 (2.86%) | 10 (8.47%) | 8 (5.71%) | 6 (3.33%) | 10 (5.56%) | 3 (1.5%) | 18 (9.89%) | 5 (3.33%) | 8 (4%) | 2 (1.04%) | 5 (2.78%) |
|  | A*3104 | 3 (1.67%) | NA | 1 (0.94%) | NA | NA | NA | NA | NA | NA | NA | NA | NA | NA | NA |
|  | A*3201 | 4 (2.22%) | 1 (0.83%) | NA | 3 (2.14%) | NA | 4 (2.86%) | 2 (1.11%) | NA | NA | NA | 6 (4%) | 8 (4%) | 7 (3.65%) | 10 (5.56%) |
|  | A*3301g | NA | 3 (2.5%) | 1 (0.94%) | 1 (0.71%) | 1 (0.85%) | 4 (2.86%) | NA | NA | NA | NA | NA | NA | 1 (0.52%) | 2 (1.11%) |
|  | A*3303g | 2 (1.11%) | 13 (10.83%) | 2 (1.89%) | 3 (2.14%) | NA | 1 (0.71%) | 15 (8.33%) | 4 (2.22%) | 12 (6%) | 19 (10.44%) | NA | NA | NA | 3 (1.67%) |
|  | A*3402 | 8 (4.44%) | 5 (4.17%) | 2 (1.89%) | 1 (0.71%) | 1 (0.85%) | NA | NA | NA | NA | NA | NA | NA | NA | NA |
|  | A*3601 | 6 (3.33%) | 17 (14.17%) | 2 (1.89%) | NA | NA | NA | NA | NA | NA | NA | NA | NA | NA | NA |
|  | A*6601g | 10 (5.56%) | NA | NA | 1 (0.71%) | NA | NA | NA | NA | NA | NA | NA | NA | NA | 1 (0.56%) |
|  | A*6602 | 7 (3.89%) | 2 (1.67%) | NA | NA | NA | NA | NA | NA | NA | NA | NA | NA | NA | NA |
|  | A*6801g | NA | 2 (1.67%) | 7 (6.6%) | 4 (2.86%) | 9 (7.63%) | 5 (3.57%) | 4 (2.22%) | 1 (0.56%) | NA | NA | 5 (3.33%) | 11 (5.5%) | 8 (4.17%) | 3 (1.67%) |
|  | A*6802 | 14 (7.78%) | 7 (5.83%) | 8 (7.55%) | 5 (3.57%) | 3 (2.54%) | 5 (3.57%) | NA | NA | NA | NA | NA | NA | NA | NA |
|  | A*6803 | NA | NA | NA | NA | 1 (0.85%) | NA | NA | NA | NA | NA | NA | NA | NA | NA |
|  | A*6901 | NA | NA | NA | NA | NA | NA | NA | NA | NA | NA | NA | 1 (0.5%) | NA | 1 (0.56%) |
|  | A*7401g | 12 (6.67%) | 5 (4.17%) | 5 (4.72%) | 1 (0.71%) | 1 (0.85%) | 1 (0.71%) | NA | 1 (0.56%) | 1 (0.5%) | NA | NA | NA | NA | NA |
|  | A*7403 | 2 (1.11%) | NA | NA | NA | NA | NA | NA | NA | NA | NA | NA | NA | NA | NA |
|  | A*8001 | 1 (0.56%) | 1 (0.83%) | 2 (1.89%) | NA | NA | 1 (0.71%) | NA | NA | NA | NA | NA | NA | NA | NA |
|  | A*9247 | NA | NA | NA | 1 (0.71%) | NA | NA | NA | NA | NA | NA | NA | NA | NA | NA |

| ***HLA Locus*** | ***Ancestry*** | ***African*** | | ***American*** | | | | ***East Asian*** | | | | ***European*** | | | |
| --- | --- | --- | --- | --- | --- | --- | --- | --- | --- | --- | --- | --- | --- | --- | --- |
|  | ***Code*** | ***LWK*** | ***YRI*** | ***ASW*** | ***CLM*** | ***MXL*** | ***PUR*** | ***CHB*** | ***CHD*** | ***CHS*** | ***JPT*** | ***CEU*** | ***FIN*** | ***GBR*** | ***TSI*** |
| ***HLA*B Alleles*** | B*0702g | 3 (1.67%) | 9 (7.5%) | 7 (6.6%) | 11 (7.86%) | 5 (4.24%) | 12 (8.57%) | 4 (2.22%) | 1 (0.56%) | 4 (2%) | 10 (5.49%) | 25 (16.67%) | 26 (13%) | 23 (11.98%) | 17 (9.44%) |
|  | B*0705g | NA | NA | NA | 1 (0.71%) | NA | 2 (1.43%) | 1 (0.56%) | 1 (0.56%) | NA | NA | NA | NA | NA | 3 (1.67%) |
|  | B*0801g | 9 (5%) | 1 (0.83%) | 1 (0.94%) | 2 (1.43%) | 3 (2.54%) | 6 (4.29%) | 2 (1.11%) | NA | 1 (0.5%) | NA | 17 (11.33%) | 14 (7%) | 17 (8.85%) | 10 (5.56%) |
|  | B*1301 | NA | NA | NA | NA | NA | NA | 7 (3.89%) | 14 (7.78%) | 15 (7.5%) | 2 (1.1%) | NA | NA | NA | NA |
|  | B*1302 | 2 (1.11%) | 3 (2.5%) | 4 (3.77%) | 2 (1.43%) | 1 (0.85%) | NA | 9 (5%) | 7 (3.89%) | 3 (1.5%) | 1 (0.55%) | 6 (4%) | 7 (3.5%) | 4 (2.08%) | 8 (4.44%) |
|  | B*1401 | NA | 1 (0.83%) | 1 (0.94%) | NA | 3 (2.54%) | NA | NA | NA | NA | NA | 2 (1.33%) | NA | 3 (1.56%) | 1 (0.56%) |
|  | B*1402 | 2 (1.11%) | 1 (0.83%) | NA | 8 (5.71%) | 9 (7.63%) | 8 (5.71%) | NA | NA | NA | NA | 1 (0.67%) | NA | 7 (3.65%) | 3 (1.67%) |
|  | B*1403 | NA | 1 (0.83%) | NA | NA | NA | NA | NA | NA | NA | NA | NA | NA | NA | NA |
|  | B*1501g | NA | NA | 1 (0.94%) | 5 (3.57%) | 3 (2.54%) | 2 (1.43%) | 10 (5.56%) | 8 (4.44%) | 7 (3.5%) | 9 (4.95%) | 14 (9.33%) | 23 (11.5%) | 5 (2.6%) | 5 (2.78%) |
|  | B*1502 | NA | NA | NA | NA | NA | NA | 4 (2.22%) | 12 (6.67%) | 8 (4%) | NA | NA | NA | NA | NA |
|  | B*1503g | 13 (7.22%) | 7 (5.83%) | 4 (3.77%) | NA | 1 (0.85%) | 3 (2.14%) | NA | NA | NA | NA | NA | NA | 1 (0.52%) | NA |
|  | B*1507 | NA | NA | NA | NA | NA | NA | 1 (0.56%) | 1 (0.56%) | NA | 4 (2.2%) | NA | NA | NA | NA |
|  | B*1509 | NA | NA | NA | NA | NA | NA | NA | NA | NA | NA | NA | NA | NA | 1 (0.56%) |
|  | B*1510 | 8 (4.44%) | 10 (8.33%) | 3 (2.83%) | NA | NA | 1 (0.71%) | NA | NA | NA | NA | 1 (0.67%) | NA | NA | NA |
|  | B*1511 | NA | NA | NA | NA | NA | NA | 1 (0.56%) | 4 (2.22%) | 2 (1%) | NA | NA | NA | NA | NA |
|  | B*1512g | NA | NA | NA | NA | NA | NA | NA | 2 (1.11%) | 1 (0.5%) | NA | NA | NA | NA | NA |
|  | B*1515 | NA | NA | NA | NA | 3 (2.54%) | NA | NA | NA | NA | NA | NA | NA | NA | NA |
|  | B*1516 | NA | 1 (0.83%) | 1 (0.94%) | 2 (1.43%) | 1 (0.85%) | 2 (1.43%) | NA | NA | NA | NA | NA | NA | NA | NA |
|  | B*1517 | 3 (1.67%) | NA | 1 (0.94%) | 1 (0.71%) | NA | NA | NA | NA | NA | NA | NA | NA | NA | 3 (1.67%) |
|  | B*1518 | NA | NA | NA | NA | NA | 1 (0.71%) | 4 (2.22%) | 2 (1.11%) | 2 (1%) | 4 (2.2%) | NA | 1 (0.5%) | NA | 1 (0.56%) |
|  | B*1520 | NA | NA | NA | NA | NA | 1 (0.71%) | NA | NA | NA | NA | NA | NA | NA | NA |
|  | B*1525 | NA | NA | NA | NA | NA | NA | NA | 1 (0.56%) | 1 (0.5%) | NA | NA | NA | NA | NA |
|  | B*1527 | NA | NA | NA | NA | NA | NA | 2 (1.11%) | 2 (1.11%) | 1 (0.5%) | NA | NA | NA | NA | NA |
|  | B*1537 | NA | NA | 1 (0.94%) | NA | NA | NA | NA | NA | NA | NA | NA | NA | NA | NA |
|  | B*1801g | 9 (5%) | 6 (5%) | 2 (1.89%) | 4 (2.86%) | 7 (5.93%) | 6 (4.29%) | NA | NA | NA | NA | 5 (3.33%) | 5 (2.5%) | 2 (1.04%) | 13 (7.22%) |
|  | B*1803 | 1 (0.56%) | NA | NA | NA | NA | NA | NA | NA | NA | NA | NA | NA | NA | NA |
|  | B*1805 | NA | NA | NA | NA | NA | 1 (0.71%) | NA | NA | NA | NA | NA | NA | NA | NA |
|  | B*1806 | NA | NA | NA | NA | 1 (0.85%) | NA | NA | NA | NA | NA | NA | NA | NA | NA |
|  | B*2703g | 1 (0.56%) | NA | NA | NA | NA | NA | NA | NA | NA | NA | 3 (2%) | NA | NA | NA |
|  | B*2704 | NA | NA | NA | NA | NA | NA | NA | 1 (0.56%) | 3 (1.5%) | 1 (0.55%) | NA | NA | NA | NA |
|  | B*2705g | NA | NA | 2 (1.89%) | 2 (1.43%) | 2 (1.69%) | 1 (0.71%) | NA | 1 (0.56%) | NA | NA | NA | 15 (7.5%) | 12 (6.25%) | 2 (1.11%) |
|  | B*2707 | NA | NA | NA | NA | NA | NA | 1 (0.56%) | NA | NA | NA | NA | NA | NA | NA |
|  | B*3501g | 8 (4.44%) | 10 (8.33%) | 5 (4.72%) | 10 (7.14%) | 6 (5.08%) | 12 (8.57%) | 6 (3.33%) | 2 (1.11%) | 8 (4%) | 14 (7.69%) | 8 (5.33%) | 33 (16.5%) | 8 (4.17%) | 9 (5%) |
|  | B*3502 | NA | NA | NA | NA | 1 (0.85%) | 4 (2.86%) | NA | NA | NA | NA | NA | NA | 1 (0.52%) | 6 (3.33%) |
|  | B*3502/3503/3504 | NA | NA | NA | NA | NA | NA | NA | NA | NA | NA | 1 (0.67%) | NA | NA | NA |
|  | B*3503g | NA | NA | 1 (0.94%) | NA | 2 (1.69%) | 4 (2.86%) | 4 (2.22%) | NA | NA | NA | 1 (0.67%) | NA | 4 (2.08%) | 7 (3.89%) |
|  | B*3505 | NA | NA | NA | 1 (0.71%) | 1 (0.85%) | NA | NA | 1 (0.56%) | NA | NA | NA | NA | NA | NA |
|  | B*3508 | NA | NA | NA | 4 (2.86%) | 2 (1.69%) | 1 (0.71%) | NA | NA | NA | NA | NA | NA | NA | 2 (1.11%) |
|  | B*3510 | NA | NA | NA | 2 (1.43%) | NA | NA | NA | NA | NA | NA | NA | NA | NA | NA |
|  | B*3511 | NA | NA | NA | NA | NA | 2 (1.43%) | NA | NA | NA | NA | NA | NA | NA | NA |
|  | B*3512 | NA | NA | NA | 4 (2.86%) | 2 (1.69%) | 1 (0.71%) | NA | NA | NA | NA | NA | NA | NA | NA |
|  | B*3514 | NA | NA | NA | NA | 2 (1.69%) | NA | NA | NA | NA | NA | NA | NA | NA | NA |
|  | B*3517 | NA | NA | NA | NA | 8 (6.78%) | NA | NA | NA | NA | NA | NA | NA | NA | NA |
|  | B*3543g | NA | NA | NA | 10 (7.14%) | NA | NA | NA | NA | NA | NA | NA | NA | NA | NA |
|  | B*3701 | NA | NA | NA | NA | NA | NA | 3 (1.67%) | 2 (1.11%) | 1 (0.5%) | 2 (1.1%) | 1 (0.67%) | 1 (0.5%) | 3 (1.56%) | 2 (1.11%) |
|  | B*3801 | NA | NA | NA | 3 (2.14%) | 2 (1.69%) | NA | 2 (1.11%) | NA | NA | NA | 1 (0.67%) | 3 (1.5%) | 3 (1.56%) | 4 (2.22%) |
|  | B*3802g | NA | NA | NA | NA | NA | NA | 4 (2.22%) | 7 (3.89%) | 4 (2%) | NA | NA | NA | NA | NA |
|  | B*3901g | NA | NA | NA | 1 (0.71%) | NA | 2 (1.43%) | 1 (0.56%) | 3 (1.67%) | 10 (5%) | 6 (3.3%) | NA | 6 (3%) | 3 (1.56%) | 1 (0.56%) |
|  | B*3902 | NA | NA | NA | NA | NA | NA | NA | NA | NA | 1 (0.55%) | NA | NA | NA | NA |
|  | B*3903 | NA | 1 (0.83%) | NA | NA | NA | NA | NA | NA | NA | NA | NA | NA | NA | NA |
|  | B*3905 | NA | NA | NA | 3 (2.14%) | 2 (1.69%) | 2 (1.43%) | NA | 1 (0.56%) | NA | NA | NA | NA | NA | NA |
|  | B*3906 | NA | NA | 1 (0.94%) | 2 (1.43%) | 6 (5.08%) | NA | NA | NA | NA | NA | 1 (0.67%) | NA | 3 (1.56%) | 3 (1.67%) |
|  | B*3908 | NA | NA | NA | 1 (0.71%) | NA | NA | NA | NA | NA | NA | NA | NA | NA | NA |
|  | B*3910 | 1 (0.56%) | 1 (0.83%) | 2 (1.89%) | NA | NA | NA | NA | NA | NA | NA | NA | NA | NA | NA |
|  | B*3911 | NA | NA | NA | 5 (3.57%) | NA | NA | NA | NA | NA | NA | NA | NA | NA | NA |
|  | B*4001g | NA | NA | 3 (2.83%) | 1 (0.71%) | NA | 4 (2.86%) | 22 (12.22%) | 28 (15.56%) | 41 (20.5%) | 9 (4.95%) | 9 (6%) | 15 (7.5%) | 15 (7.81%) | 2 (1.11%) |
|  | B*4002g | NA | NA | 1 (0.94%) | 6 (4.29%) | 4 (3.39%) | 4 (2.86%) | 4 (2.22%) | 2 (1.11%) | 4 (2%) | 20 (10.99%) | 3 (2%) | 5 (2.5%) | 2 (1.04%) | NA |
|  | B*4004 | NA | NA | NA | 3 (2.14%) | NA | 1 (0.71%) | NA | NA | NA | NA | NA | NA | NA | NA |
|  | B*4005 | NA | NA | NA | NA | 1 (0.85%) | NA | NA | NA | NA | NA | NA | NA | NA | NA |
|  | B*4006 | NA | NA | NA | NA | NA | NA | 5 (2.78%) | 6 (3.33%) | 2 (1%) | 9 (4.95%) | NA | NA | NA | NA |
|  | B*4012 | 2 (1.11%) | NA | NA | NA | NA | NA | NA | NA | NA | NA | NA | NA | NA | NA |
|  | B*4020 | NA | NA | NA | NA | 1 (0.85%) | NA | NA | NA | NA | NA | NA | NA | NA | NA |
|  | B*4101 | 3 (1.67%) | NA | NA | 1 (0.71%) | 2 (1.69%) | NA | NA | NA | NA | NA | 1 (0.67%) | 2 (1%) | 2 (1.04%) | 1 (0.56%) |
|  | B*4102 | NA | NA | 1 (0.94%) | 1 (0.71%) | 1 (0.85%) | NA | NA | NA | NA | NA | 1 (0.67%) | NA | NA | 3 (1.67%) |
|  | B*4104 | NA | 1 (0.83%) | NA | NA | NA | NA | NA | NA | NA | NA | NA | NA | NA | NA |
|  | B*4201 | 12 (6.67%) | 8 (6.67%) | 7 (6.6%) | 1 (0.71%) | NA | 1 (0.71%) | NA | NA | NA | NA | NA | NA | NA | NA |
|  | B*4202 | NA | 1 (0.83%) | NA | 1 (0.71%) | 1 (0.85%) | NA | NA | NA | NA | NA | NA | NA | NA | NA |
|  | B*44 | NA | NA | NA | NA | NA | NA | NA | NA | NA | NA | 1 (0.67%) | NA | NA | NA |
|  | B*4402g | NA | NA | 2 (1.89%) | 6 (4.29%) | 1 (0.85%) | 7 (5%) | 3 (1.67%) | NA | NA | 1 (0.55%) | 20 (13.33%) | 16 (8%) | 23 (11.98%) | 10 (5.56%) |
|  | B*4403 | 3 (1.67%) | 2 (1.67%) | 7 (6.6%) | 8 (5.71%) | 4 (3.39%) | 15 (10.71%) | 2 (1.11%) | 2 (1.11%) | 1 (0.5%) | 16 (8.79%) | 6 (4%) | 2 (1%) | 14 (7.29%) | 9 (5%) |
|  | B*4405 | NA | NA | 1 (0.94%) | NA | 1 (0.85%) | NA | NA | NA | NA | NA | NA | NA | NA | NA |
|  | B*4501g | 17 (9.44%) | 2 (1.67%) | 3 (2.83%) | NA | 2 (1.69%) | 3 (2.14%) | NA | NA | NA | NA | NA | NA | 3 (1.56%) | NA |
|  | B*4601g | NA | NA | NA | NA | NA | NA | 21 (11.67%) | 19 (10.56%) | 37 (18.5%) | 10 (5.49%) | NA | NA | NA | NA |
|  | B*4701 | NA | NA | NA | NA | 1 (0.85%) | NA | NA | NA | NA | NA | NA | 3 (1.5%) | NA | NA |
|  | B*4801g | NA | NA | 1 (0.94%) | NA | 6 (5.08%) | NA | 7 (3.89%) | 2 (1.11%) | 4 (2%) | 4 (2.2%) | NA | NA | NA | NA |
|  | B*4802 | NA | NA | NA | NA | NA | 1 (0.71%) | NA | NA | NA | NA | NA | NA | NA | NA |
|  | B*4803 | NA | NA | NA | NA | 1 (0.85%) | NA | NA | NA | 1 (0.5%) | NA | NA | NA | NA | NA |
|  | B*4805 | 1 (0.56%) | NA | NA | NA | NA | NA | NA | NA | NA | NA | NA | NA | NA | NA |
|  | B*4901 | 1 (0.56%) | 6 (5%) | 2 (1.89%) | 5 (3.57%) | NA | 1 (0.71%) | NA | NA | NA | NA | 1 (0.67%) | NA | NA | 6 (3.33%) |
|  | B*5001 | NA | NA | 2 (1.89%) | 2 (1.43%) | 1 (0.85%) | 2 (1.43%) | 1 (0.56%) | 1 (0.56%) | NA | NA | 3 (2%) | NA | NA | 3 (1.67%) |
|  | B*5101g | 8 (4.44%) | 5 (4.17%) | 2 (1.89%) | 8 (5.71%) | 6 (5.08%) | 8 (5.71%) | 12 (6.67%) | 11 (6.11%) | 10 (5%) | 14 (7.69%) | 4 (2.67%) | 7 (3.5%) | 9 (4.69%) | 24 (13.33%) |
|  | B*5102g | NA | NA | NA | NA | 2 (1.69%) | NA | 1 (0.56%) | 3 (1.67%) | 2 (1%) | NA | NA | NA | NA | NA |
|  | B*5108 | NA | NA | NA | NA | NA | NA | NA | NA | 1 (0.5%) | NA | NA | NA | NA | NA |
|  | B*5123 | NA | NA | NA | NA | 1 (0.85%) | NA | NA | NA | NA | NA | NA | NA | NA | NA |
|  | B*5201g | 1 (0.56%) | 10 (8.33%) | NA | NA | 3 (2.54%) | 5 (3.57%) | 6 (3.33%) | 2 (1.11%) | 1 (0.5%) | 22 (12.09%) | 2 (1.33%) | NA | NA | 1 (0.56%) |
|  | B*5301 | 22 (12.22%) | 19 (15.83%) | 19 (17.92%) | 4 (2.86%) | NA | 2 (1.43%) | NA | NA | NA | NA | NA | NA | 1 (0.52%) | 2 (1.11%) |
|  | B*5401g | NA | NA | NA | NA | NA | NA | 8 (4.44%) | 10 (5.56%) | 3 (1.5%) | 8 (4.4%) | NA | NA | NA | NA |
|  | B*5501 | NA | NA | 1 (0.94%) | NA | 1 (0.85%) | NA | NA | NA | NA | NA | 1 (0.67%) | 3 (1.5%) | 5 (2.6%) | 6 (3.33%) |
|  | B*5501/5502 | NA | NA | NA | NA | NA | NA | NA | NA | NA | NA | 1 (0.67%) | NA | NA | NA |
|  | B*5502 | NA | NA | NA | NA | NA | NA | 2 (1.11%) | 7 (3.89%) | 6 (3%) | 2 (1.1%) | NA | NA | NA | NA |
|  | B*5512 | NA | NA | NA | NA | NA | NA | NA | 1 (0.56%) | NA | NA | NA | NA | NA | NA |
|  | B*5601g | NA | 1 (0.83%) | NA | NA | 1 (0.85%) | 4 (2.86%) | 1 (0.56%) | 2 (1.11%) | 2 (1%) | 2 (1.1%) | 1 (0.67%) | 6 (3%) | 3 (1.56%) | NA |
|  | B*5701 | NA | NA | 1 (0.94%) | 4 (2.86%) | 2 (1.69%) | 3 (2.14%) | 1 (0.56%) | 2 (1.11%) | 3 (1.5%) | NA | 9 (6%) | 6 (3%) | 14 (7.29%) | 7 (3.89%) |
|  | B*5702 | 1 (0.56%) | 1 (0.83%) | NA | NA | NA | NA | NA | NA | NA | NA | NA | NA | NA | 1 (0.56%) |
|  | B*5703 | 4 (2.22%) | 3 (2.5%) | 3 (2.83%) | 1 (0.71%) | NA | 2 (1.43%) | NA | 1 (0.56%) | NA | NA | NA | NA | NA | NA |
|  | B*5801g | 11 (6.11%) | 7 (5.83%) | 6 (5.66%) | 2 (1.43%) | NA | 2 (1.43%) | 14 (7.78%) | 8 (4.44%) | 10 (5%) | 3 (1.65%) | NA | NA | 2 (1.04%) | 4 (2.22%) |
|  | B*5802 | 18 (10%) | NA | 2 (1.89%) | 1 (0.71%) | 1 (0.85%) | NA | NA | NA | NA | NA | NA | NA | NA | NA |
|  | B*5901 | NA | NA | NA | NA | NA | NA | 1 (0.56%) | NA | NA | 4 (2.2%) | NA | NA | NA | NA |
|  | B*6701 | NA | NA | NA | NA | NA | NA | 2 (1.11%) | NA | 1 (0.5%) | 4 (2.2%) | NA | 1 (0.5%) | NA | NA |
|  | B*7301 | 2 (1.11%) | NA | NA | NA | NA | NA | NA | NA | NA | NA | NA | NA | NA | NA |
|  | B*7801 | NA | 1 (0.83%) | 1 (0.94%) | NA | NA | 1 (0.71%) | NA | NA | NA | NA | NA | NA | NA | NA |
|  | B*8101g | 12 (6.67%) | 1 (0.83%) | 4 (3.77%) | 1 (0.71%) | NA | NA | 1 (0.56%) | NA | NA | NA | NA | NA | NA | NA |
|  | B*8201 | NA | NA | NA | NA | 1 (0.85%) | NA | NA | NA | NA | NA | NA | NA | NA | NA |
|  | B*8202 | 2 (1.11%) | NA | NA | NA | NA | NA | NA | NA | NA | NA | NA | NA | NA | NA |

| ***HLA Locus*** | ***Ancestry*** | ***African*** | | ***American*** | | | | ***East Asian*** | | | | ***European*** | | | |
| --- | --- | --- | --- | --- | --- | --- | --- | --- | --- | --- | --- | --- | --- | --- | --- |
|  | ***Code*** | ***LWK*** | ***YRI*** | ***ASW*** | ***CLM*** | ***MXL*** | ***PUR*** | ***CHB*** | ***CHD*** | ***CHS*** | ***JPT*** | ***CEU*** | ***FIN*** | ***GBR*** | ***TSI*** |
| ***HLA-C Alleles*** | C*0102g | NA | 1 (0.83%) | 2 (1.89%) | 15 (10.71%) | 10 (8.47%) | 5 (3.57%) | 29 (16.11%) | 32 (17.78%) | 40 (20%) | 24 (13.19%) | 2 (1.33%) | 11 (5.5%) | 9 (4.69%) | 3 (1.67%) |
|  | C*0103g | NA | NA | NA | NA | NA | NA | 1 (0.56%) | 1 (0.56%) | 1 (0.5%) | 2 (1.1%) | NA | NA | NA | NA |
|  | C*0108 | NA | NA | NA | NA | NA | NA | NA | 1 (0.56%) | NA | NA | NA | NA | NA | NA |
|  | C*0140 | NA | NA | NA | NA | NA | NA | NA | 1 (0.56%) | NA | NA | NA | NA | NA | NA |
|  | C*0202 | 1 (0.56%) | 11 (9.17%) | 1 (0.94%) | 1 (0.71%) | 1 (0.85%) | 3 (2.14%) | NA | 1 (0.56%) | NA | NA | 4 (2.67%) | 12 (6%) | 10 (5.21%) | 4 (2.22%) |
|  | C*0210 | 10 (5.56%) | NA | 6 (5.66%) | NA | 1 (0.85%) | 3 (2.14%) | NA | NA | NA | NA | NA | NA | 1 (0.52%) | NA |
|  | C*0302 | 6 (3.33%) | 5 (4.17%) | 2 (1.89%) | NA | 1 (0.85%) | NA | 14 (7.78%) | 6 (3.33%) | 9 (4.5%) | 3 (1.65%) | NA | NA | NA | 2 (1.11%) |
|  | C*0303/0304 | NA | NA | NA | NA | NA | NA | NA | NA | NA | NA | 1 (0.67%) | NA | NA | NA |
|  | C*0303g | NA | NA | 2 (1.89%) | 4 (2.86%) | 5 (4.24%) | 2 (1.43%) | 11 (6.11%) | 8 (4.44%) | 16 (8%) | 25 (13.74%) | 9 (6%) | 18 (9%) | 8 (4.17%) | 9 (5%) |
|  | C*0304 | 9 (5%) | NA | 5 (4.72%) | 7 (5%) | 7 (5.93%) | 10 (7.14%) | 14 (7.78%) | 23 (12.78%) | 34 (17%) | 25 (13.74%) | 13 (8.67%) | 27 (13.5%) | 17 (8.85%) | 3 (1.67%) |
|  | C*0305 | NA | NA | 1 (0.94%) | 5 (3.57%) | NA | 2 (1.43%) | NA | NA | NA | NA | NA | NA | NA | NA |
|  | C*0336 | NA | NA | NA | NA | NA | NA | NA | NA | 1 (0.5%) | NA | NA | NA | NA | NA |
|  | C*0356 | NA | NA | NA | NA | NA | NA | NA | 1 (0.56%) | NA | NA | NA | NA | NA | NA |
|  | C*0401g | 34 (18.89%) | 30 (25%) | 26 (24.53%) | 26 (18.57%) | 24 (20.34%) | 27 (19.29%) | 12 (6.67%) | 8 (4.44%) | 8 (4%) | 4 (2.2%) | 13 (8.67%) | 36 (18%) | 15 (7.81%) | 27 (15%) |
|  | C*0403 | NA | NA | NA | NA | NA | NA | NA | 4 (2.22%) | 2 (1%) | NA | NA | NA | NA | NA |
|  | C*0404 | 2 (1.11%) | NA | NA | NA | NA | NA | NA | NA | NA | NA | NA | NA | NA | NA |
|  | C*0407 | NA | NA | NA | NA | NA | NA | NA | NA | NA | NA | NA | NA | NA | NA |
|  | C*0501g | 3 (1.67%) | 1 (0.83%) | 4 (3.77%) | 12 (8.57%) | 5 (4.24%) | 10 (7.14%) | 3 (1.67%) | NA | NA | 1 (0.55%) | 17 (11.33%) | 12 (6%) | 21 (10.94%) | 14 (7.78%) |
|  | C*0509 | NA | NA | NA | NA | NA | NA | NA | NA | NA | NA | NA | NA | NA | NA |
|  | C*0602 | 29 (16.11%) | 1 (0.83%) | 6 (5.66%) | 7 (5%) | 7 (5.93%) | 6 (4.29%) | 14 (7.78%) | 13 (7.22%) | 7 (3.5%) | 3 (1.65%) | 20 (13.33%) | 17 (8.5%) | 23 (11.98%) | 19 (10.56%) |
|  | C*0701g | 14 (7.78%) | 16 (13.33%) | 10 (9.43%) | 12 (8.57%) | 6 (5.08%) | 13 (9.29%) | 1 (0.56%) | 1 (0.56%) | NA | NA | 19 (12.67%) | 18 (9%) | 20 (10.42%) | 26 (14.44%) |
|  | C*0702g | 7 (3.89%) | 7 (5.83%) | 8 (7.55%) | 21 (15%) | 13 (11.02%) | 14 (10%) | 30 (16.67%) | 37 (20.56%) | 46 (23%) | 26 (14.29%) | 26 (17.33%) | 27 (13.5%) | 27 (14.06%) | 18 (10%) |
|  | C*0704g | 10 (5.56%) | NA | 2 (1.89%) | NA | NA | 1 (0.71%) | 1 (0.56%) | 1 (0.56%) | NA | 2 (1.1%) | 6 (4%) | 6 (3%) | 3 (1.56%) | 1 (0.56%) |
|  | C*0801g | NA | NA | NA | NA | 10 (8.47%) | NA | 19 (10.56%) | 19 (10.56%) | 15 (7.5%) | 14 (7.69%) | NA | NA | NA | NA |
|  | C*0802 | 2 (1.11%) | 6 (5%) | 1 (0.94%) | 8 (5.71%) | 12 (10.17%) | 7 (5%) | NA | NA | NA | NA | 3 (2%) | NA | 10 (5.21%) | 4 (2.22%) |
|  | C*0803 | NA | NA | NA | NA | NA | NA | 1 (0.56%) | NA | 2 (1%) | NA | NA | NA | NA | NA |
|  | C*0804 | 3 (1.67%) | 6 (5%) | 9 (8.49%) | 1 (0.71%) | NA | NA | NA | NA | NA | NA | NA | NA | NA | NA |
|  | C*1202 | 1 (0.56%) | NA | NA | NA | 1 (0.85%) | 2 (1.43%) | 6 (3.33%) | 4 (2.22%) | 2 (1%) | 23 (12.64%) | 2 (1.33%) | NA | NA | 1 (0.56%) |
|  | C*1203 | 3 (1.67%) | 1 (0.83%) | 3 (2.83%) | 4 (2.86%) | 5 (4.24%) | 6 (4.29%) | 6 (3.33%) | 4 (2.22%) | 2 (1%) | NA | 3 (2%) | 7 (3.5%) | 6 (3.12%) | 18 (10%) |
|  | C*14 | NA | NA | NA | NA | NA | NA | 1 (0.56%) | NA | NA | NA | NA | NA | NA | NA |
|  | C*1402 | NA | 1 (0.83%) | 1 (0.94%) | 2 (1.43%) | 1 (0.85%) | 2 (1.43%) | 8 (4.44%) | 8 (4.44%) | 7 (3.5%) | 13 (7.14%) | NA | 2 (1%) | 3 (1.56%) | 7 (3.89%) |
|  | C*1403 | 1 (0.56%) | NA | NA | NA | NA | NA | NA | NA | NA | 16 (8.79%) | NA | NA | NA | NA |
|  | C*15 | NA | NA | NA | NA | NA | NA | NA | NA | NA | NA | 1 (0.67%) | NA | NA | NA |
|  | C*1502g | NA | NA | 1 (0.94%) | 4 (2.86%) | 1 (0.85%) | 6 (4.29%) | 9 (5%) | 5 (2.78%) | 7 (3.5%) | 1 (0.55%) | 5 (3.33%) | 4 (2%) | 4 (2.08%) | 9 (5%) |
|  | C*1504 | NA | NA | NA | NA | NA | NA | NA | 1 (0.56%) | NA | NA | NA | NA | NA | NA |
|  | C*1505 | 3 (1.67%) | 2 (1.67%) | NA | 1 (0.71%) | NA | 3 (2.14%) | NA | 1 (0.56%) | NA | NA | NA | NA | NA | 3 (1.67%) |
|  | C*1601 | 20 (11.11%) | 20 (16.67%) | 7 (6.6%) | 6 (4.29%) | 5 (4.24%) | 15 (10.71%) | NA | NA | NA | NA | 5 (3.33%) | 2 (1%) | 11 (5.73%) | 5 (2.78%) |
|  | C*1602 | NA | NA | NA | NA | 1 (0.85%) | 1 (0.71%) | NA | NA | 1 (0.5%) | NA | NA | NA | 1 (0.52%) | 1 (0.56%) |
|  | C*1604 | NA | NA | NA | NA | NA | 1 (0.71%) | NA | NA | NA | NA | NA | NA | 1 (0.52%) | 1 (0.56%) |
|  | C*1701g | 14 (7.78%) | 10 (8.33%) | 8 (7.55%) | 4 (2.86%) | 2 (1.69%) | 1 (0.71%) | NA | NA | NA | NA | 1 (0.67%) | 1 (0.5%) | 2 (1.04%) | 4 (2.22%) |
|  | C*1801g | 8 (4.44%) | 2 (1.67%) | 1 (0.94%) | NA | NA | NA | NA | NA | NA | NA | NA | NA | NA | 1 (0.56%) |

| ***HLA Locus*** | ***Ancestry*** | ***African*** | | ***American*** | | | | ***East Asian*** | | | | ***European*** | | | |
| --- | --- | --- | --- | --- | --- | --- | --- | --- | --- | --- | --- | --- | --- | --- | --- |
|  | ***Code*** | ***LWK*** | ***YRI*** | ***ASW*** | ***CLM*** | ***MXL*** | ***PUR*** | ***CHB*** | ***CHD*** | ***CHS*** | ***JPT*** | ***CEU*** | ***FIN*** | ***GBR*** | ***TSI*** |
| ***HLA-DRB1 Alleles*** | DRB1*0101 | 3 (1.67%) | 2 (1.67%) | 6 (5.66%) | 16 (11.43%) | 6 (5.08%) | 6 (4.29%) | 5 (2.78%) | 1 (0.56%) | 4 (2%) | 9 (4.95%) | 12 (8%) | 40 (20%) | 20 (10.42%) | 10 (5.56%) |
|  | DRB1*0102g | 9 (5%) | 4 (3.33%) | 2 (1.89%) | 3 (2.14%) | 6 (5.08%) | 7 (5%) | NA | NA | NA | NA | NA | NA | 1 (0.52%) | 4 (2.22%) |
|  | DRB1*0103 | NA | NA | 1 (0.94%) | 3 (2.14%) | 1 (0.85%) | 1 (0.71%) | NA | NA | NA | NA | NA | NA | NA | NA |
|  | DRB1*0301 | 10 (5.56%) | 11 (9.17%) | 5 (4.72%) | 6 (4.29%) | 11 (9.32%) | 15 (10.71%) | 12 (6.67%) | 6 (3.33%) | 6 (3%) | NA | 14 (9.33%) | 13 (6.5%) | 20 (10.42%) | 19 (10.56%) |
|  | DRB1*0302 | 15 (8.33%) | 10 (8.33%) | 5 (4.72%) | 3 (2.14%) | NA | 5 (3.57%) | NA | NA | NA | NA | NA | NA | NA | NA |
|  | DRB1*0401 | 1 (0.56%) | NA | 3 (2.83%) | NA | NA | 2 (1.43%) | 2 (1.11%) | 2 (1.11%) | NA | 2 (1.1%) | 17 (11.33%) | 17 (8.5%) | 19 (9.9%) | 4 (2.22%) |
|  | DRB1*0402 | NA | NA | NA | 1 (0.71%) | 1 (0.85%) | 3 (2.14%) | NA | NA | NA | NA | 1 (0.67%) | 1 (0.5%) | NA | 1 (0.56%) |
|  | DRB1*0403 | NA | 1 (0.83%) | NA | 4 (2.86%) | 3 (2.54%) | 3 (2.14%) | 4 (2.22%) | 1 (0.56%) | 1 (0.5%) | 9 (4.95%) | 1 (0.67%) | 1 (0.5%) | 3 (1.56%) | NA |
|  | DRB1*0403/0406 | NA | NA | NA | NA | NA | NA | NA | NA | NA | NA | 1 (0.67%) | NA | NA | NA |
|  | DRB1*0404 | NA | NA | 3 (2.83%) | 5 (3.57%) | 7 (5.93%) | 5 (3.57%) | 2 (1.11%) | 1 (0.56%) | 2 (1%) | NA | 16 (10.67%) | 7 (3.5%) | 16 (8.33%) | 5 (2.78%) |
|  | DRB1*0405 | NA | 2 (1.67%) | 1 (0.94%) | 2 (1.43%) | 2 (1.69%) | 4 (2.86%) | 12 (6.67%) | 9 (5%) | 7 (3.5%) | 19 (10.44%) | NA | NA | 1 (0.52%) | NA |
|  | DRB1*0406 | NA | NA | NA | NA | NA | NA | 1 (0.56%) | 3 (1.67%) | 7 (3.5%) | 3 (1.65%) | NA | NA | NA | 1 (0.56%) |
|  | DRB1*0407 | NA | NA | 3 (2.83%) | 10 (7.14%) | 6 (5.08%) | 1 (0.71%) | NA | NA | 2 (1%) | NA | 2 (1.33%) | 1 (0.5%) | 2 (1.04%) | 2 (1.11%) |
|  | DRB1*0408 | NA | NA | NA | 1 (0.71%) | NA | NA | NA | NA | NA | NA | NA | 3 (1.5%) | NA | NA |
|  | DRB1*0410 | NA | NA | NA | 1 (0.71%) | NA | NA | 1 (0.56%) | NA | NA | 4 (2.2%) | NA | NA | NA | NA |
|  | DRB1*0411 | NA | NA | NA | 5 (3.57%) | 5 (4.24%) | 7 (5%) | NA | NA | NA | NA | NA | NA | NA | NA |
|  | DRB1*0468 | NA | NA | NA | NA | NA | NA | NA | 1 (0.56%) | NA | NA | NA | NA | NA | NA |
|  | DRB1*0701 | 4 (2.22%) | 13 (10.83%) | 7 (6.6%) | 11 (7.86%) | 9 (7.63%) | 20 (14.29%) | 15 (8.33%) | 12 (6.67%) | 8 (4%) | 2 (1.1%) | 22 (14.67%) | 15 (7.5%) | 28 (14.58%) | 27 (15%) |
|  | DRB1*0711 | NA | NA | NA | 1 (0.71%) | NA | NA | NA | NA | NA | NA | NA | NA | NA | NA |
|  | DRB1*0801 | NA | NA | NA | 1 (0.71%) | 2 (1.69%) | 2 (1.43%) | 2 (1.11%) | NA | NA | 2 (1.1%) | 4 (2.67%) | 25 (12.5%) | 8 (4.17%) | 5 (2.78%) |
|  | DRB1*0802 | NA | NA | 1 (0.94%) | 7 (5%) | 15 (12.71%) | 3 (2.14%) | 1 (0.56%) | NA | NA | 11 (6.04%) | NA | 1 (0.5%) | NA | 2 (1.11%) |
|  | DRB1*0803 | NA | NA | NA | NA | NA | NA | 16 (8.89%) | 13 (7.22%) | 24 (12%) | 8 (4.4%) | NA | NA | NA | 1 (0.56%) |
|  | DRB1*0804 | 13 (7.22%) | 9 (7.5%) | 11 (10.38%) | 1 (0.71%) | 1 (0.85%) | NA | NA | NA | NA | NA | NA | NA | NA | 3 (1.67%) |
|  | DRB1*0806 | NA | NA | 3 (2.83%) | 1 (0.71%) | NA | NA | NA | NA | NA | NA | NA | NA | NA | NA |
|  | DRB1*0812 | NA | NA | NA | NA | NA | NA | NA | NA | NA | 1 (0.55%) | NA | NA | NA | NA |
|  | DRB1*0901 | 3 (1.67%) | 2 (1.67%) | 3 (2.83%) | 3 (2.14%) | NA | 1 (0.71%) | 28 (15.56%) | 46 (25.56%) | 35 (17.5%) | 27 (14.84%) | NA | 6 (3%) | 2 (1.04%) | NA |
|  | DRB1*1001 | 5 (2.78%) | 1 (0.83%) | 4 (3.77%) | 3 (2.14%) | 1 (0.85%) | 1 (0.71%) | 4 (2.22%) | 2 (1.11%) | 1 (0.5%) | 2 (1.1%) | 2 (1.33%) | 3 (1.5%) | 1 (0.52%) | 3 (1.67%) |
|  | DRB1*1101 | 30 (16.67%) | 7 (5.83%) | 4 (3.77%) | 2 (1.43%) | 3 (2.54%) | 10 (7.14%) | 9 (5%) | 6 (3.33%) | 20 (10%) | 3 (1.65%) | 4 (2.67%) | 7 (3.5%) | 6 (3.12%) | 17 (9.44%) |
|  | DRB1*1102 | 15 (8.33%) | 4 (3.33%) | 4 (3.77%) | 2 (1.43%) | 3 (2.54%) | 2 (1.43%) | NA | NA | NA | NA | NA | NA | 2 (1.04%) | NA |
|  | DRB1*1103 | NA | NA | NA | NA | 1 (0.85%) | 1 (0.71%) | NA | NA | NA | NA | 1 (0.67%) | NA | 1 (0.52%) | 2 (1.11%) |
|  | DRB1*1104 | 1 (0.56%) | 6 (5%) | 1 (0.94%) | 1 (0.71%) | 3 (2.54%) | 4 (2.86%) | 1 (0.56%) | 1 (0.56%) | NA | NA | 1 (0.67%) | 1 (0.5%) | 2 (1.04%) | 13 (7.22%) |
|  | DRB1*1201g | 12 (6.67%) | 2 (1.67%) | 3 (2.83%) | 1 (0.71%) | 1 (0.85%) | 3 (2.14%) | 10 (5.56%) | 9 (5%) | 6 (3%) | 7 (3.85%) | 2 (1.33%) | 6 (3%) | 3 (1.56%) | 4 (2.22%) |
|  | DRB1*1202 | NA | NA | NA | NA | NA | NA | 10 (5.56%) | 21 (11.67%) | 15 (7.5%) | 1 (0.55%) | NA | NA | NA | NA |
|  | DRB1*1301 | 10 (5.56%) | 13 (10.83%) | 7 (6.6%) | 9 (6.43%) | 2 (1.69%) | 11 (7.86%) | 3 (1.67%) | NA | 1 (0.5%) | 1 (0.55%) | 5 (3.33%) | 15 (7.5%) | 7 (3.65%) | 10 (5.56%) |
|  | DRB1*1302 | 14 (7.78%) | 6 (5%) | 8 (7.55%) | 7 (5%) | NA | 5 (3.57%) | 5 (2.78%) | 1 (0.56%) | 1 (0.5%) | 18 (9.89%) | 3 (2%) | 7 (3.5%) | 9 (4.69%) | 10 (5.56%) |
|  | DRB1*1303 | 2 (1.11%) | 9 (7.5%) | 7 (6.6%) | 5 (3.57%) | NA | 2 (1.43%) | NA | NA | NA | NA | 1 (0.67%) | NA | 4 (2.08%) | 2 (1.11%) |
|  | DRB1*1304 | NA | NA | 1 (0.94%) | NA | 1 (0.85%) | NA | NA | NA | NA | NA | NA | NA | NA | NA |
|  | DRB1*1305 | NA | NA | NA | NA | NA | NA | NA | NA | NA | NA | 1 (0.67%) | NA | NA | NA |
|  | DRB1*1312 | NA | NA | NA | NA | NA | NA | 1 (0.56%) | 3 (1.67%) | 3 (1.5%) | NA | NA | NA | NA | NA |
|  | DRB1*1321 | NA | NA | NA | NA | NA | NA | NA | NA | NA | NA | NA | NA | NA | 1 (0.56%) |
|  | DRB1*1327 | NA | 1 (0.83%) | NA | NA | NA | NA | NA | NA | NA | NA | NA | NA | NA | NA |
|  | DRB1*1335 | NA | 1 (0.83%) | NA | NA | NA | NA | NA | NA | NA | NA | NA | NA | NA | NA |
|  | DRB1*1401g | 2 (1.11%) | 3 (2.5%) | 2 (1.89%) | 1 (0.71%) | 1 (0.85%) | 2 (1.43%) | 5 (2.78%) | 6 (3.33%) | 10 (5%) | 7 (3.85%) | 4 (2.67%) | 1 (0.5%) | 7 (3.65%) | 9 (5%) |
|  | DRB1*1402 | NA | NA | 1 (0.94%) | 5 (3.57%) | 4 (3.39%) | 1 (0.71%) | 1 (0.56%) | NA | NA | NA | NA | NA | NA | NA |
|  | DRB1*1403 | NA | NA | NA | NA | NA | NA | NA | NA | 1 (0.5%) | 5 (2.75%) | NA | NA | NA | NA |
|  | DRB1*1404 | NA | NA | NA | NA | NA | 1 (0.71%) | NA | 2 (1.11%) | 1 (0.5%) | NA | 1 (0.67%) | NA | NA | NA |
|  | DRB1*1405 | NA | NA | NA | NA | NA | NA | 3 (1.67%) | 5 (2.78%) | 5 (2.5%) | 2 (1.1%) | NA | NA | NA | NA |
|  | DRB1*1406 | NA | NA | NA | NA | 8 (6.78%) | NA | NA | NA | NA | NA | NA | NA | NA | NA |
|  | DRB1*1407 | NA | NA | NA | NA | NA | NA | 1 (0.56%) | NA | 1 (0.5%) | NA | NA | NA | NA | NA |
|  | DRB1*1444 | NA | NA | NA | NA | NA | NA | 1 (0.56%) | NA | NA | NA | NA | NA | NA | NA |
|  | DRB1*1501 | NA | 1 (0.83%) | 2 (1.89%) | 8 (5.71%) | 7 (5.93%) | 6 (4.29%) | 16 (8.89%) | 17 (9.44%) | 31 (15.5%) | 16 (8.79%) | 33 (22%) | 28 (14%) | 30 (15.62%) | 17 (9.44%) |
|  | DRB1*1502 | 1 (0.56%) | NA | NA | 1 (0.71%) | 2 (1.69%) | 1 (0.71%) | 5 (2.78%) | 3 (1.67%) | 1 (0.5%) | 23 (12.64%) | 1 (0.67%) | NA | NA | 2 (1.11%) |
|  | DRB1*1503 | 30 (16.67%) | 11 (9.17%) | 6 (5.66%) | 3 (2.14%) | NA | 1 (0.71%) | NA | NA | NA | NA | NA | NA | NA | NA |
|  | DRB1*1601 | NA | NA | NA | 2 (1.43%) | NA | 3 (2.14%) | NA | NA | NA | NA | 1 (0.67%) | 2 (1%) | NA | 5 (2.78%) |
|  | DRB1*1602 | NA | 1 (0.83%) | 2 (1.89%) | 5 (3.57%) | 6 (5.08%) | 1 (0.71%) | 4 (2.22%) | 9 (5%) | 7 (3.5%) | NA | NA | NA | NA | 1 (0.56%) |

| ***HLA Locus*** | ***Ancestry*** | ***African*** | | ***American*** | | | | ***East Asian*** | | | | ***European*** | | | |
| --- | --- | --- | --- | --- | --- | --- | --- | --- | --- | --- | --- | --- | --- | --- | --- |
|  | ***Code*** | ***LWK*** | ***YRI*** | ***ASW*** | ***CLM*** | ***MXL*** | ***PUR*** | ***CHB*** | ***CHD*** | ***CHS*** | ***JPT*** | ***CEU*** | ***FIN*** | ***GBR*** | ***TSI*** |
| ***HLA-DQB1 Alleles*** | DQB1*0201g | 18 (10%) | 33 (27.5%) | 20 (18.87%) | 20 (14.29%) | 19 (16.1%) | 33 (23.57%) | 25 (13.89%) | 16 (8.89%) | 11 (5.5%) | 2 (1.1%) | 29 (19.33%) | 21 (10.5%) | 37 (19.27%) | 37 (20.56%) |
|  | DQB1*0301g | 40 (22.22%) | 21 (17.5%) | 23 (21.7%) | 22 (15.71%) | 30 (25.42%) | 23 (16.43%) | 32 (17.78%) | 40 (22.22%) | 44 (22%) | 15 (8.24%) | 22 (14.67%) | 21 (10.5%) | 33 (17.19%) | 43 (23.89%) |
|  | DQB1*0302 | 2 (1.11%) | 2 (1.67%) | 9 (8.49%) | 24 (17.14%) | 22 (18.64%) | 20 (14.29%) | 9 (5%) | 8 (4.44%) | 13 (6.5%) | 20 (10.99%) | 26 (17.33%) | 23 (11.5%) | 25 (13.02%) | 8 (4.44%) |
|  | DQB1*0303 | 1 (0.56%) | NA | 1 (0.94%) | 4 (2.86%) | 2 (1.69%) | 3 (2.14%) | 30 (16.67%) | 48 (26.67%) | 38 (19%) | 30 (16.48%) | 7 (4.67%) | 13 (6.5%) | 12 (6.25%) | 8 (4.44%) |
|  | DQB1*0304 | NA | NA | NA | NA | 1 (0.85%) | 1 (0.71%) | NA | NA | NA | NA | NA | NA | 1 (0.52%) | NA |
|  | DQB1*04 | NA | NA | NA | NA | NA | NA | NA | NA | NA | 2 (1.1%) | NA | NA | NA | NA |
|  | DQB1*0401 | NA | NA | NA | NA | NA | NA | 11 (6.11%) | 7 (3.89%) | 5 (2.5%) | 17 (9.34%) | NA | NA | NA | NA |
|  | DQB1*0402 | 15 (8.33%) | 13 (10.83%) | 7 (6.6%) | 13 (9.29%) | 18 (15.25%) | 12 (8.57%) | 3 (1.67%) | NA | 1 (0.5%) | 8 (4.4%) | 4 (2.67%) | 26 (13%) | 8 (4.17%) | 12 (6.67%) |
|  | DQB1*0501 | 43 (23.89%) | 15 (12.5%) | 17 (16.04%) | 26 (18.57%) | 14 (11.86%) | 15 (10.71%) | 10 (5.56%) | 5 (2.78%) | 5 (2.5%) | 11 (6.04%) | 14 (9.33%) | 43 (21.5%) | 22 (11.46%) | 15 (8.33%) |
|  | DQB1*0502 | NA | 7 (5.83%) | 4 (3.77%) | 5 (3.57%) | NA | 3 (2.14%) | 10 (5.56%) | 15 (8.33%) | 19 (9.5%) | 2 (1.1%) | 1 (0.67%) | 2 (1%) | NA | 10 (5.56%) |
|  | DQB1*0503 | 1 (0.56%) | 2 (1.67%) | NA | 2 (1.43%) | 1 (0.85%) | 3 (2.14%) | 6 (3.33%) | 11 (6.11%) | 9 (4.5%) | 7 (3.85%) | 5 (3.33%) | 2 (1%) | 7 (3.65%) | 9 (5%) |
|  | DQB1*0504 | NA | NA | 1 (0.94%) | NA | NA | NA | NA | NA | NA | NA | NA | NA | NA | 1 (0.56%) |
|  | DQB1*0601 | 1 (0.56%) | NA | NA | 1 (0.71%) | 2 (1.69%) | 1 (0.71%) | 23 (12.78%) | 19 (10.56%) | 35 (17.5%) | 35 (19.23%) | 1 (0.67%) | NA | NA | 2 (1.11%) |
|  | DQB1*0602 | 46 (25.56%) | 12 (10%) | 12 (11.32%) | 9 (6.43%) | 7 (5.93%) | 9 (6.43%) | 13 (7.22%) | 9 (5%) | 18 (9%) | 14 (7.69%) | 33 (22%) | 26 (13%) | 29 (15.1%) | 13 (7.22%) |
|  | DQB1*0603 | 2 (1.11%) | 8 (6.67%) | 1 (0.94%) | 8 (5.71%) | 2 (1.69%) | 11 (7.86%) | 3 (1.67%) | NA | 1 (0.5%) | 1 (0.55%) | 5 (3.33%) | 16 (8%) | 9 (4.69%) | 12 (6.67%) |
|  | DQB1*0604g | 4 (2.22%) | 2 (1.67%) | 3 (2.83%) | 5 (3.57%) | NA | 4 (2.86%) | 3 (1.67%) | NA | NA | 15 (8.24%) | 3 (2%) | 7 (3.5%) | 6 (3.12%) | 6 (3.33%) |
|  | DQB1*0605 | NA | 5 (4.17%) | NA | NA | NA | NA | NA | NA | NA | 1 (0.55%) | NA | NA | NA | NA |
|  | DQB1*0609 | 7 (3.89%) | NA | 8 (7.55%) | 1 (0.71%) | NA | 2 (1.43%) | 2 (1.11%) | 1 (0.56%) | 1 (0.5%) | 2 (1.1%) | NA | NA | 3 (1.56%) | 4 (2.22%) |
|  | DQB1*0610 | NA | NA | NA | NA | NA | NA | NA | 1 (0.56%) | NA | NA | NA | NA | NA | NA |
