# Supplementary material for: HLA Diversity in the 1000 Genomes Dataset
Source: PLoS One. 2014 Jul 2;9(7):e97282. doi: 10.1371/journal.pone.0097282 (PMC4079705; doi:10.1371/journal.pone.0097282)
Supplement: Table S3 — HLA alleles grouped by similarities in the antigen recognition site. (DOCX) [file pone.0097282.s007.docx]

**Table S3 HLA alleles grouped by similarities in the antigen recognition site.**

| *Current Name | *Equivalent as of Aug2011 |
| --- | --- |
| A*0101g | A*01010101/01010102N/0104N/0122N/0132/0134N/0137 |
| A*0201g | A*02010101/02010102L/02010103/020108/020111/020114/020115/020121/0209/0243N/0266/0275/0283N/0289/0297/9232/9234/9240 |
| A*0205g | A*020501/9279 |
| A*0206g | A*020601/9226 |
| A*0207g | A*0207/0215N |
| A*0211g | A*0211/0269 |
| A*0217 | A*021701/021702 |
| A*0222g | A*022201/9204 |
| A*0301g | A*03010101/03010102N/03010103/030107/0320/0321N/0326/0337/0345 |
| A*1101g | A*110101/1121N |
| A*1102 | A*110201/110203 |
| A*2301g | A*230101/2307N/2317/2318/2320 |
| A*2402g | A*24020101/24020102L/240203/240210/240213/2409N/2411N/2440N/2476/2479/2483N |
| A*2403g | A*240301/2433 |
| A*2501g | A*250101/2507 |
| A*2601g | A*260101/260107/2624/2626 |
| A*2901g | A*29010101/29010102N |
| A*3001g | A*300101/300102/3024 |
| A*3002 | A*300201/300202 |
| A*3101g | A*310102/3114N/3123 |
| A*3201 | A*320101/320102 |
| A*3303g | A*330301/330303/3315/3325 |
| A*6601g | A*6601/6608 |
| A*6801 | A*680101/680107 |
| A*6801g | A*680102/6811N/6833 |
| A*680201 | A*68020101/68020102/68020103 |
| A*7401g | A*7401/7402 |

| *Current Name | *Equivalent as of Aug2011 |
| --- | --- |
| B*0702g | B*070201/070206/070209/0744/0749N/0758/0759/0761 |
| B*0705g | B*070501/0706 |
| B*0801g | B*080101/0819N |
| B*1302 | B*130201/130205 |
| B*1501g | B*15010101/15010102N/150106/150107/9502/9504/9540/9546 |
| B*1503g | B*150301/9503 |
| B*1512g | B*1512/1519 |
| B*151701 | B*15170101/15170102 |
| B*1801g | B*180101/180103/1817N |
| B*2705g | B*270502/270504/2713 |
| B*3501g | B*350101/350103/3540N/3542/3557/3594 |
| B*3503g | B*350301/3570 |
| B*3543g | B*3543/3567/3579 |
| B*3802g | B*380201/3818 |
| B*3901g | B*39010101/39010102L/390103/3946 |
| B*4001g | B*400101/400102/4055 |
| B*4002g | B*400201/4056/4097 |
| B*400601 | B*40060101/40060102 |
| B*4402g | B*44020101/44020102S/4419N/4427/4466 |
| B*4403 | B*440301/440303/440304 |
| B*4501g | B*4501/4507 |
| B*4601g | B*460101/4615N |
| B*470101 | B*47010101/47010102 |
| B*4801g | B*480101/4809 |
| B*5101g | B*510101/510105/510107/5111N/5130/5132/5148/5151 |
| B*5201g | B*520101/5207 |
| B*5401g | B*5401/5417 |
| B*5501 | B*550101/550103 |
| B*5601g | B*5601/5624 |
| B*5801g | B*580101/5811 |
| B*8101g | B*8101/8102/8103 |

| *Current Name | *Equivalent as of Aug2011 |
| --- | --- |
| C*0102g | C*010201/010202/010203/010204/010205/010206/010207/010208/010209/0125 |
| C*0103g | C*0103/0124 |
| C*0302 | C*030201/030202/030203 |
| C*0303g | C*030301/0320N |
| C*0304 | C*030401/030403 |
| C*0401g | C*04010101/04010102/04010103/0409N/0428/0430/0441 |
| C*0501g | C*05010101/05010102/050104/050105/0503 |
| C*0602 | C*06020101/06020102/060203 |
| C*0701g | C*070101/070102/070109/0706/0718/0752 |
| C*0702g | C*07020101/07020102/07020103/0750/0766/0774 |
| C*0704g | C*070401/0711 |
| C*0801g | C*080101/0820/0822/0824 |
| C*1202 | C*120201/120202 |
| C*1203 | C*12030101/12030102/120306 |
| C*1502g | C*150201/1513 |
| C*1505 | C*150501/150502/150503 |
| C*1701g | C*1701/1702/1703 |
| C*1801g | C*1801/1802 |

| *Current Name | *Equivalent as of Aug2011 |
| --- | --- |
| DRB1*030101 | DRB1*03010101/03010102 |
| DRB1*0406 | DRB1*040601/040602 |
| DRB1*070101 | DRB1*07010101/07010102 |
| DRB1*0801 | DRB1*080101/080103 |
| DRB1*1101 | DRB1*110101/110108 |
| DRB1*1201g | DRB1*120101/1206/1210/1217 |
| DRB1*1401g | DRB1*140101/1454 |
| DRB1*150101 | DRB1*15010101/15010102 |
| DRB1*150301 | DRB1*15030101/15030102 |

| *Current Name | *Equivalent as of Aug2011 |
| --- | --- |
| DQB1*0201g | DQB1*020101/0202/0204 |
| DQB1*0301g | DQB1*030101/030104/0309/0319/0321/0322/0324 |
| DQB1*0601 | DQB1*060101/060103 |
| DQB1*0604g | DQB1*060401/0634 |
