# Supplementary material for: HLA Diversity in the 1000 Genomes Dataset
Source: PLoS One. 2014 Jul 2;9(7):e97282. doi: 10.1371/journal.pone.0097282 (PMC4079705; doi:10.1371/journal.pone.0097282)
Supplement: Table S4 — The most frequent ancestry specific HLA haplotypes. (DOCX) [file pone.0097282.s008.docx]

**Tables S4 The most frequent ancestry specific HLA haplotypes**

| Ancestry | Selection of frequent HLA ancestral haplotypes | also frequent in |  | Ancestry | Selection of frequent HLA ancestral haplotypes | also frequent in |
| --- | --- | --- | --- | --- | --- | --- |
| European | A*0101~B*0801~DRB1*0301 |  |  | Asians | A*3303~B*5801~DRB1*0301 |  |
|  | A*0301~B*0702~DRB1*1501 |  |  |  | A*0207~B*4601~DRB1*0901 |  |
|  | A*0201~B*4402~DRB1*0401 |  |  |  | A*3303~B*4403~DRB1*0701 |  |
|  | A*0201~B*0702~DRB1*1501 |  |  |  | A*3001~B*1302~DRB1*0701 | Europeans |
|  | A*2902~B*4403~DRB1*0701 | Hispanics |  |  | A*3303~B*5801~DRB1*1302 |  |
|  | A*0201~B*1501~DRB1*0401 |  |  |  | A*1101~B*1502~DRB1*1202 |  |
|  | A*0101~B*5701~DRB1*0701 |  |  |  | A*2402~B*5201~DRB1*1502 |  |
|  | A*0301~B*3501~DRB1*0101 |  |  |  | A*0101~B*5701~DRB1*0701 | Europeans |
|  | A*3001~B*1302~DRB1*0701 | Asians |  |  | A*3303~B*4403~DRB1*1302 |  |
|  | A*0201~B*0801~DRB1*0301 |  |  |  | A*0101~B*3701~DRB1*1001 |  |
|  | A*0201~B*4001~DRB1*1302 |  |  | Africans | A*3001~B*4201~DRB1*0302 |  |
|  | A*0201~B*5701~DRB1*0701 |  |  |  | A*0101~B*0801~DRB1*0301 | Europeans |
| Hispanics | A*2902~B*4403~DRB1*0701 |  |  |  | A*6801~B*5802~DRB1*1201 |  |
|  | A*0101~B*0801~DRB1*0301 | Europeans |  |  | A*6802~B*1510~DRB1*0301 |  |
|  | A*0301~B*0702~DRB1*1501 | Europeans |  |  | A*3303~B*5301~DRB1*0804 |  |
|  | A*3002~B*1801~DRB1*0301 |  |  |  | A*3601~B*5301~DRB1*1101 |  |
|  | A*3301~B*1402~DRB1*0102 |  |  |  | A*0301~B*0702~DRB1*1501 | Hispanics |
|  | A*6803~B*3905~DRB1*0407 |  |  |  | A*3402~B*4403~DRB1*1503 |  |
|  | A*2301~B*4403~DRB1*0701 |  |  |  | A*2902~B*4403~DRB1*0702 | Hispanics |
|  | A*2402~B*3906~DRB1*1406 |  |  |  | A*0201~B*4402~DRB1*0401 | Europeans |
|  | A*0201~B*0702~DRB1*1501 | Europeans |  |  |  |  |
|  | A*0206~B*3905~DRB1*0407 |  |  |  |  |  |
