# Supplementary material for: HLA Diversity in the 1000 Genomes Dataset
Source: PLoS One. 2014 Jul 2;9(7):e97282. doi: 10.1371/journal.pone.0097282 (PMC4079705; doi:10.1371/journal.pone.0097282)
Supplement: Table S5 — Variants associated with frequent haplotypes in Europeans. (DOCX) [file pone.0097282.s009.docx]

**Table S5: Variants associated with frequent haplotypes in Europeans**

| **Frequent HLA Haplotype** | **Variant frequently associated (r2>0.6)** |
| --- | --- |
| **A*0101~B*0801~DRB1*0301** | rs3135293 rs3132378 rs2230683 rs13206458 rs3135318 rs3118361 rs3130892 rs3130895 rs3131068 rs3131069 rs3131071 rs3130844 rs3131072 rs3130845 rs3130847 rs3131076 rs148696809 rs3130885 rs3130837 rs3129791 rs3130890 rs3131079 rs3129794 rs3130891 rs3130893 rs3130894 rs3131081 rs3130896 rs3135317 rs3129797 rs3129798 rs3129799 rs3117146 rs3129800 rs9278145 rs3117141 rs3117143 rs3131085 rs3129788 rs3129178 rs3129112 rs3130773 rs3130777 rs3130715 rs3130746 rs3129173 rs3116830 rs3130831 rs3129104 rs3117339 rs3117337 rs3117326 rs3130834 rs3129682 rs9257800 rs442694 rs1535039 rs2523443 rs2143668 rs2206853 rs2746150 rs1234844 rs1233494 rs1233491 rs1233489 rs1233488 rs2523431 rs408623 rs389419 rs404240 rs413670 rs1235162 rs1233393 rs1233385 rs3131856 rs3131894 rs3116813 rs3098023 rs3094146 rs3115631 rs8321 rs9261290 rs2023473 rs2523978 rs2844789 rs1970 rs1573296 rs2157678 rs3094078 rs3129830 rs3094069 rs3094628 rs3130355 rs3129812 rs3132619 rs3094717 rs3132616 rs3094024 rs3130241 rs9262126 rs9262135 rs9262143 rs3132600 rs886420 rs3131781 rs1632863 rs1634716 rs1634721 rs1634726 rs3130544 rs3132510 rs3093958 rs3131618 rs3130614 rs3130612 rs1800628 rs3132450 rs3117582 rs3117581 rs3132449 rs9267531 rs9267539 rs9267544 rs9267549 rs3131383 rs3101018 rs3131381 rs3131380 rs3132445 rs3132443 rs3130484 rs3117573 rs3131379 rs3117574 rs3131378 rs3117575 rs3117577 rs3115672 rs3101017 rs3115671 rs3130490 rs3130491 rs915652 rs915651 rs2607014 rs2736425 rs2763980 rs681331 rs625421 rs553414 rs3130476 rs3130478 rs3130679 rs599707 rs9267574 rs9267578 rs501942 rs1265905 rs1270942 rs389884 rs433061 rs1150757 rs1150756 rs1150753 rs1150752 rs1269852 rs3130288 rs3132941 rs3130297 rs9267986 rs3132953 rs146576271 rs3132971 rs3115556 rs9268144 rs7775397 rs9268152 rs9268158 rs9268177 rs9268208 rs9268219 rs116667074 rs7751715 rs9268235 rs4392743 rs1265757 rs1265754 rs926593 rs2849018 rs910051 rs2395149 rs2894254 rs3129950 rs3129966 rs3129843 rs3129855 rs3129856 rs3135394 rs7383481 rs7748925 rs3135383 rs9268813 rs7763411 |
| **A*0201~B*4402~DRB1*0401** | rs41268932 rs8192574 |
| **A*2902~B*4403~DRB1*0701** | rs17438538 rs138004456 rs259948 rs2158287 rs140895602 |
| **A*0201~B*1501~DRB1*0401** | rs2254620 rs2248477 |
| **A*0301~B*3501~DRB1*0101** | rs114878174 rs116462901 rs114060326 |
| **A*3001~B*1302~DRB1*0701** | rs117584100 rs117614998 rs117763669 rs17188127 rs118169754 rs117660163 rs117626418 rs117560270 |

| **Frequent HLA Haplotype** | **Variant frequently associated (r2>0.6)** |
| --- | --- |
| **A*6801~B*5802~DRB1*1201** | rs139769355 rs140935623 rs142502724 rs139078823 rs188494350 rs190632432 rs188223233 rs185605280 rs192670335 rs146732044 rs149977238 rs145723731 rs138357895 rs143635849 rs146313629 rs141927933 rs192240318 rs143303179 rs146331717 |
| **A*6802~B*1510~DRB1*0301** | rs149179383 30585632 30860027 rs189718524 rs116571463 rs190868814 rs192648574 rs114646793 |
| **A*2402~B*3906~DRB1*1406** | rs183314533 rs182263994 rs145527047 rs146464979 rs140555963 rs187395094 rs187032832 rs141014788 rs147917883 rs146450193 rs144675567 rs141939372 rs181516751 rs147299635 rs143509564 rs2022080 rs146147481 rs184627290 rs191921146 rs192020142 rs187082223 rs145281491 rs141247523 rs144414718 rs139981571 rs139987643 rs146494367 rs148241446 rs183475257 rs150256027 |
| **A*3303~B*5801~DRB1*0301** | rs77041685 rs74290517 rs79793531 rs77570173 rs75155700 rs79208225 31948194 rs77633711 rs76252543 rs75123148 rs76340303 rs77466974 rs76146382 rs142995350 |
| **A*3303~B*5801~DRB1*1302** | rs139931346 rs117160266 |
| **A*1101~B*1502~DRB1*1202** | rs191236154 rs182944420 rs183636239 |
| **A*3303~B*4403~DRB1*1302** | rs117952863 rs3757334 rs118105893 rs117636370 rs116910299 rs117322208 rs137877109 rs3778623 rs117569516 rs3757335 rs41543014 rs17875374 rs117804857 rs117383541 rs117803875 rs118120376 rs117437606 rs117093916 rs118124047 rs116877062 rs117631086 rs117687301 rs118121140 rs117764398 rs118038522 rs140942506 rs187281546 rs118052715 rs140984763 rs117248408 rs117107378 rs117644051 rs147666549 rs147733073 rs117587759 rs140773477 rs117767616 rs111887699 rs146580867 rs117116885 rs117968416 rs116915508 rs112070079 rs117484768 rs144772989 rs118114439 rs117598591 rs143395858 rs138687400 rs117576077 rs118073417 rs117722286 |
| **A*0101~B*3701~DRB1*1001** | rs150454006 rs145191873 |
